# Dramatic decline of *Sargassum* in the north Sargasso Sea since 2015

**DOI:** 10.1038/s41561-025-01863-5

**Published:** 2025-12-04

**Authors:** Yingjun Zhang, Brian B. Barnes, Deborah S. Goodwin, Amy N. S. Siuda, Jeffrey M. Schell, Dennis J. McGillicuddy, Brian E. Lapointe, Lin Qi, Chuanmin Hu

**Affiliations:** 1 College of Marine Science, University of South Florida, St. Petersburg, FL USA; 2 Sea Education Association, Woods Hole, MA USA; 3 Eckerd College, St. Petersburg, FL USA; 4 Woods Hole Oceanographic Institution, Woods Hole, MA USA; 5 Harbor Branch Oceanographic Institute, Florida Atlantic University, Fort Pierce, FL USA; 6 NOAA Center for Satellite Applications and Research, College Park, MD USA; 7 Global Science & Technology Inc., Greenbelt, MD USA

**Keywords:** Marine biology, Ocean sciences

## Abstract

The Sargasso Sea, at the centre of the North Atlantic subtropical gyre, draws its name from the endemic floating brown macroalgae, *Sargassum*, which provides shelter and habitat for life in the pelagic zone. In 2011, the *Sargassum* footprint expanded to include the Great Atlantic *Sargassum* Belt in the tropical Atlantic, but little is known about how *Sargassum* in the Sargasso Sea changed thereafter. Here we use satellite and in situ data to show that *Sargassum* in the north Sargasso Sea has declined dramatically since 2015. Accompanying this decline is a disruption in local *Sargassum* seasonal growth cycles, whereby the previously consistent fall-to-winter north Sargasso Sea biomass maxima have shifted to spring-to-summer peaks that mirror those of the Great Atlantic *Sargassum* Belt—a result of advection from this latter region. We posit that the north Sargasso Sea decline is due to reduced *Sargassum* supply from a historical Gulf of Mexico source region, possibly attributable to increasing sea surface temperatures and more frequent marine heatwaves in the Gulf of Mexico. Together, proliferation in the Great Atlantic *Sargassum* Belt and decline in the north Sargasso Sea may represent the beginnings of a regime shift in *Sargassum* distribution.

## Abstract (web summary)

*Sargassum* biomass in the north Sargasso Sea declined drastically since 2015, co-occurring with related reductions in the northwest Gulf of Mexico and an expansion of the Great Atlantic *Sargassum* Belt, according to in situ and satellite observations.

## Main

**Fig. 1:**
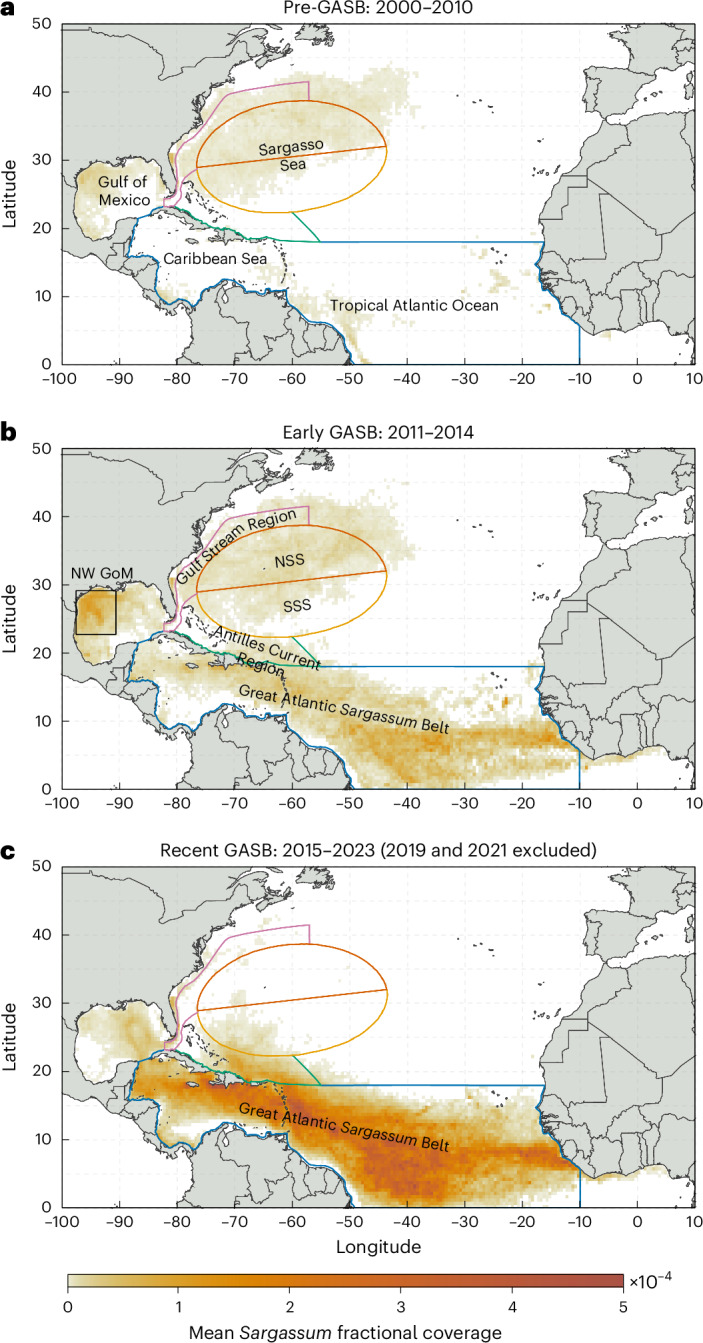
Distribution of pelagic *Sargassum* in the North Atlantic Ocean. **a**–**c**, Distribution of pelagic *Sargassum* in the North Atlantic Ocean during years 2000–2010 (**a**), 2011–2014 (**b**) and 2015–2023 (excluding calendar years 2019 and 2021) (**c**), with colour scale denoting mean fractional coverage (1 × 10^−4^ = 0.01%). The location of the SS is adapted from ref. ^49^ and outlined with an ellipse, which is separated into the north Sargasso Sea (NSS) and south Sargasso Sea (SSS)^50^. Six regions enclosed by coloured outlines (labelled in **b**, with NW GoM = northwestern Gulf of Mexico) were selected to analyse *Safrgassum* time series. For completeness, the Gulf of Guinea (east of 10° W) is included in the maps here but excluded in other maps due to negligible *Sargassum* in this region. GASB, Great Atlantic *Sargassum* Belt. Basemaps from Climate Data Toolbox^51^.

**Fig. 2:**
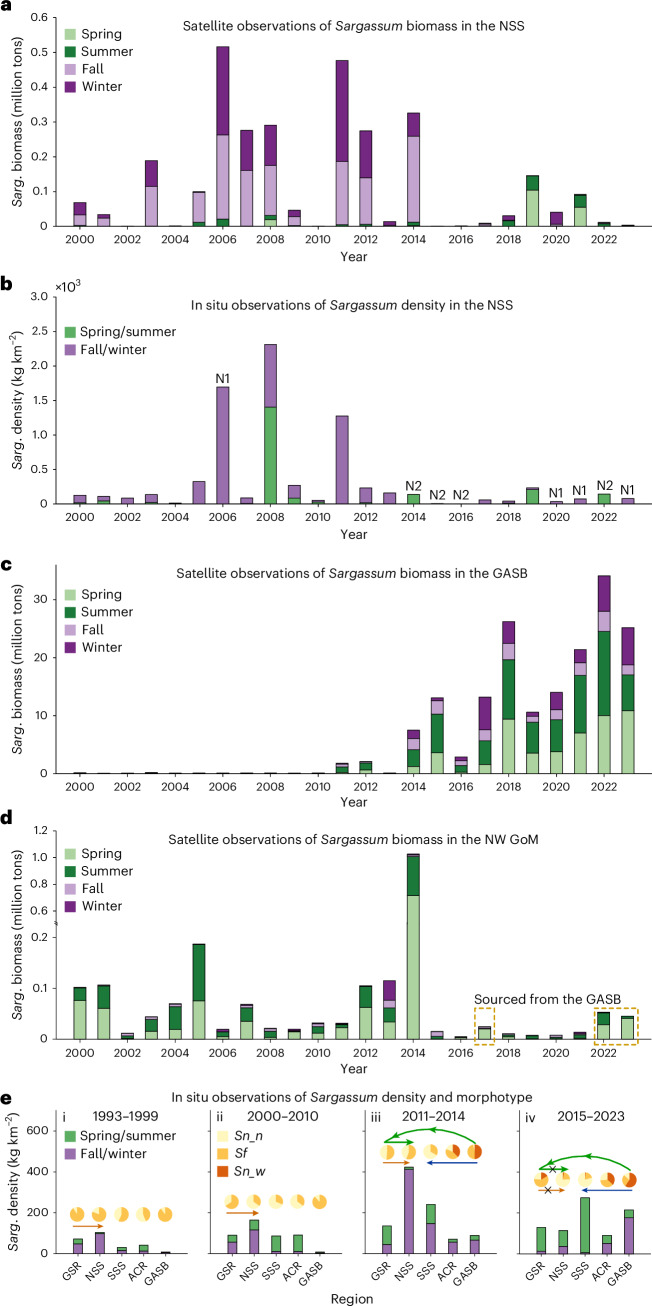
Long-term changes in *Sargassum* biomass, density and morphotypes. **a**,**c**,**d**, Satellite-derived mean *Sargassum* biomass by region: NSS (**a**), GASB (**c**) and NW GoM (**d**). **b**,**e**, In situ weighted mean *Sargassum* densities in NSS (**b**) and regional weighted means (**e**) across four time periods. In **b**, ‘N1’ and ‘N2’ indicate no in situ data for spring/summer and fall/winter, respectively. Pie charts in **e** show relative abundance of each morphotype as proportions of total *Sargassum* density. *Sn_n*, *Sf* and *Sn_w* represent *Sargassum natans* var. *natans*, *fluitans* and *natans* var. *wingei*. Arrows show transport pathways connecting regions (Pathway 1, orange; 2, green; and 3, blue; Fig. 4). Figure 1 provides regional boundary definitions for the NSS, GASB, NW GoM, Gulf Stream Region (GSR), Antilles Current Region (ACR) and SSS. The *y*-axis break in **d** is to accommodate the high *Sargassum* biomass in 2014. *Sarg.*, *Sargassum*.

**Extended Data Fig. 1:**
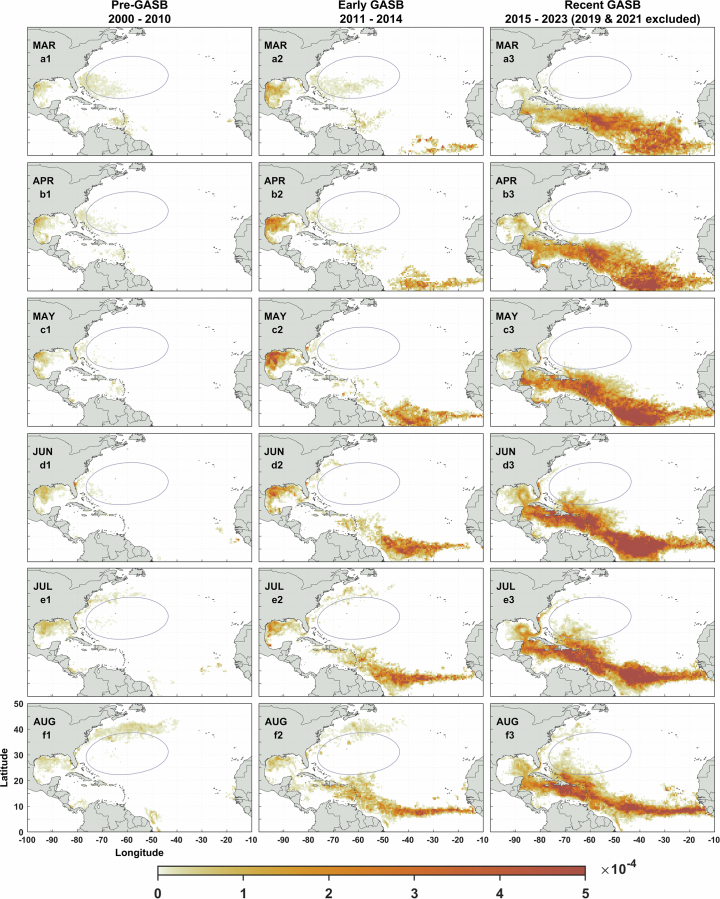
Monthly climatological distributions of *Sargassum* areal density from March through August in the Gulf of Mexico, Caribbean Sea, and the North Atlantic. The left, middle, and right panels are shown for the pre- Great Atlantic *Sargassum* Belt (GASB) era (2000–2010), early GASB period (2011–2014), and recent GASB era (2015–2023, excluding calendar years 2019 and 2021), respectively. In each subplot, the gray ellipse indicates the location of the SS, and the color scale indicates the fractional coverage of *Sargassum* (for example, 1×10^−4^ = 0.01%) in each 0.5^o^ grid. Panels identified by row (March to August corresponding to letters **a**–**f**) and column (1, pre-GASB era; 2, early GASB period; 3, recent GASB era). Basemaps from Climate Data Toolbox^51^.

**Extended Data Fig. 2:**
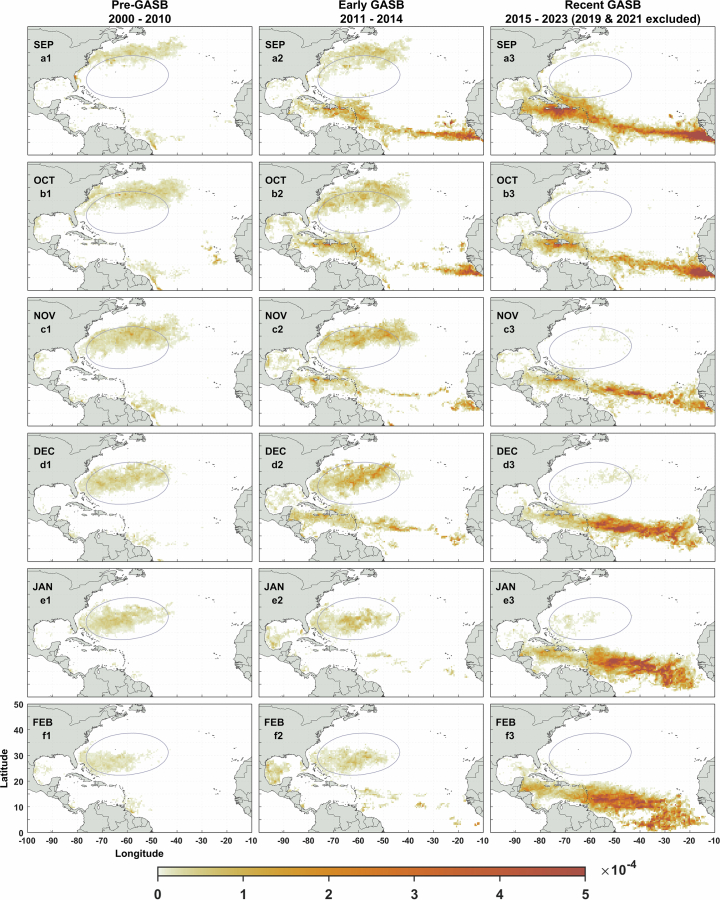
Monthly climatological distributions of *Sargassum* areal density from September through February in the Gulf of Mexico, Caribbean Sea, and the North Atlantic. The left, middle, and right panels are shown for the pre- Great Atlantic *Sargassum* Belt (GASB) era (2000–2010), early GASB period (2011–2014), and recent GASB era (2015–2023, excluding calendar years 2019 and 2021), respectively. In each subplot, the gray ellipse indicates the location of the SS, and the color scale indicates the fractional coverage of *Sargassum* (for example, 1×10^−4^ = 0.01%) in each 0.5^o^ grid. Panels identified by row (September to February corresponding to letters **a**–**f**) and column (1, pre-GASB era; 2, early GASB period; 3, recent GASB era). Basemaps from Climate Data Toolbox^51^.

Located in the subtropical North Atlantic Ocean, the Sargasso Sea (SS; Fig. 1) is bounded by ocean currents and named for its abundant holopelagic *Sargassum*. Integral to the biology and ecology of this region, *Sargassum* provides essential shelter and habitat for a diverse range of marine life^1–3^. For this reason, aggregations of *Sargassum* are called ‘golden floating rainforests’^4^. Historically, most *Sargassum* in the Atlantic Ocean was found in the SS^5,6^, although it was also reported with substantial abundance in the Gulf of Mexico (GoM) and the Caribbean Sea (CS) and sporadically documented cast ashore on islands near West Africa^7^. The predominant *Sargassum* morphotypes in the SS are *S. natans* var. *natans* (hereafter *Sn_n*) and *S. fluitans* (*Sf*; Fig. 2e)^5,6,8–11^, with SS biomass until 2010 most abundant during fall/winter^12,13^ (Extended Data Figs. 1 and 2b1–e1; correspondingly, ‘year’ in this work indicates ecological year in the SS: March to February, unless otherwise noted).

In 2011, however, large amounts of *Sargassum* were found in the CS^14^, later determined to be part of the Great Atlantic *Sargassum* Belt (GASB^15^; Fig. 1b,c). The GASB has formed every year since (except 2013), encompassing the entire tropical Atlantic where waters are warmer and more nutrient rich than the SS. In the early GASB years, *S. natans* var. *wingei* (*Sn_w*) dominated^9^ (Fig. 2eiii), a morphotype that was historically rare in the North Atlantic^5^ (Fig. 2ei,ii). More recently, all three morphotypes (*Sn_n, Sf*, and *Sn_w*) have been observed in the GASB^16,17^ (Fig. 2eiv). Since GASB discovery, numerous studies have attempted to understand the reasons behind its formation^18–20^ and its impacts on ocean ecology, carbon fixation, fisheries, coastal beach environments, tourism, economy and human health^21–29^.

With focus on the GASB, however, detailed investigation of SS *Sargassum* dynamics was lacking. Historic in situ observations documented high interannual variability in SS *Sargassum*^12,13^, which originally raised scientific questions about population decline^30^. Here, using satellite and in situ observations, we show substantial changes in *Sargassum* abundance and transport throughout the North Atlantic, including dramatically declined *Sargassum* in the North Sargasso Sea (NSS), particularly during the fall/winter seasons when it used to be sufficiently abundant for satellite detection (0.2% coverage in a pixel^31^). We assert that recent changes to the longstanding steady state, manifested through Atlantic-wide alterations in abundance, distribution, connectivity pathways and morphotype composition, may represent the beginnings of a regime shift for holopelagic *Sargassum*.

## Long-term changes and seasonality

Before the GASB (2000–2010; the ‘pre-GASB era’), satellite-derived *Sargassum* was mostly found in the SS, followed by the GoM and CS (Fig. 1a), consistent with historical field-based observations^5,9,12^. During the initial GASB years (2011–2014; ‘early GASB period’), *Sargassum* distribution patterns in the SS remained unchanged, despite more *Sargassum* being found in the GASB (Fig. 1b). By contrast, in recent years (2015–2023; ‘recent GASB era’), as the GASB expanded substantially, *Sargassum* in the NSS has nearly vanished (Fig. 1c). Exceptions are two anomalous years (2019 and 2021) during which *Sargassum* abundance peaked in regions and at times with no analogue in the satellite record (detailed below).

Through the pre-GASB era, satellite data indicated high interannual variability in NSS *Sargassum* abundance (Fig. 2a). During the early GASB period, slightly higher average abundances were observed in the NSS, although neither the variance nor the mean abundance was significantly different from those of the pre-GASB era. Additionally, the NSS *Sargassum* population appeared to originate from the northwestern GoM in both periods (detailed below). In 2015, however, NSS *Sargassum* abundance declined and has never returned to prior levels. Specifically, satellite data show mean abundance (± 1 standard deviation) of 0.175 ± 0.176 million tons in 2000–2014, compared to 0.014 ± 0.016 in 2015–2023 (*t* = 2.83; *p* = 0.011; Fig. 2a). Similarly, in situ *Sargassum* weighted mean densities declined from 203 to 113 kg km^−2^ (Fig. 2b), although comparable temporal statistical analyses are not possible with weighted means.

**Fig. 3:**
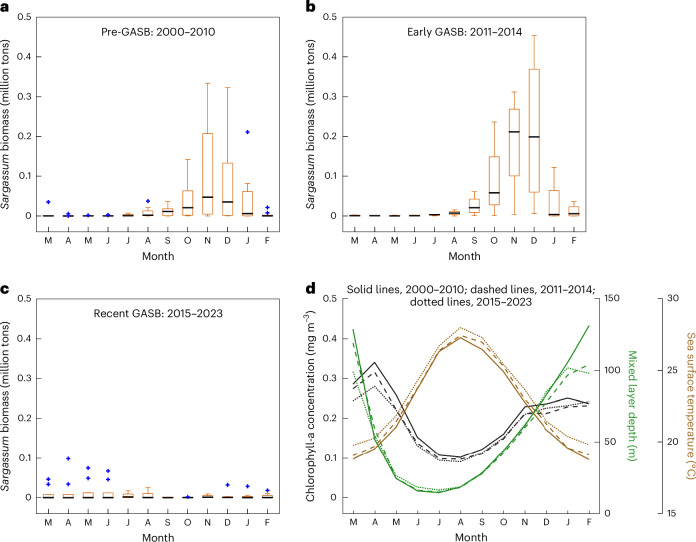
Seasonality of *Sargassum* biomass and environmental conditions in the NSS. **a**–**d**, Seasonality of satellite-derived *Sargassum* biomass (**a**–**c**) and environmental conditions (**d**) in the NSS for three periods: 2000–2010 (*n* = 11; **a**), 2011–2014 (*n* = 4; **b**) and 2015–2023 (*n* = 9; **c**). Each box in **a**–**c** is bounded by the first and third quartiles for monthly *Sargassum* biomass during the respective time period, with the median shown as a black line. Whiskers extend to the most extreme values not considered outliers (defined as 1.5 times the interquartile range from the first or third quartile, annotated as blue ‘+’). Note that the outliers in **c** all occurred in 2019 and 2021, which are discussed separately as their contributions to the SS are not in the typical fall/winter months, and their bloom initiation locations are also different.

Notably, during the pre-GASB era, NSS *Sargassum* peaked in the fall/winter months, with minimum abundance in the spring (Fig. 3a). During the early GASB period, abundance increased in the fall/winter months, yet the seasonality remained the same (Fig. 3b and Extended Data Figs. 1 and 2). In contrast, during the recent GASB era, not only did the total abundance decrease significantly from previous years, but seasonality shifted to spring/summer highs (Fig. 3c and Extended Data Figs. 1 and 2), matching that of the GASB (Fig. 2c). Furthermore, except in 2019 and 2021, nearly all NSS satellite-detected *Sargassum* during this period was restricted to a small area near the boundary with the South Sargasso Sea (SSS; Fig. 1c).

Oceanic conditions (sea surface temperature (SST), mixed layer depth (MLD) and chlorophyll-a concentration) during these three periods also showed clear seasonality (Fig. 3d). Some patterns (for example, high SST in the fall and deeper MLD in the winter; Supplementary Fig. 1) might explain NSS *Sargassum* seasonality during 2000–2014. However, oceanic conditions remained relatively stable in all years (Fig. 3d), thus these local environmental factors probably do not explain the NSS *Sargassum* decline during the recent GASB era.

## Source regions and transport pathways

**Fig. 4:**
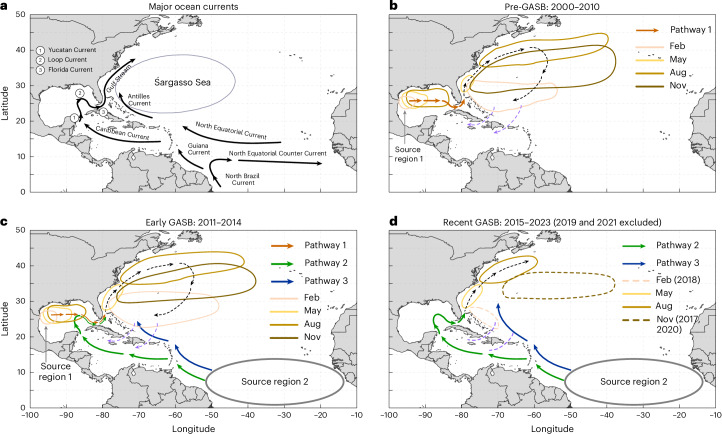
Major ocean currents, *Sargassum* sources and transport pathways. **a**–**d**, Major ocean currents (**a**) and *Sargassum* sources and transport pathways (**b**–**d**) during the years 2000–2010 (**b**), 2011–2014 (**c**) and 2015–2023 (excluding calendar years 2019 and 2021) (**d**). The dashed black lines with arrows in **b**–**d** illustrate *Sargassum* movement directions, while *Sargassum* footprints in several climatological months are indicated by coloured solid outlines. Solid orange, green and blue lines with arrows in **b**–**d** indicate satellite-observed *Sargassum* transport pathways. The dashed outlines in **d** indicate the footprint of infrequent *Sargassum* aggregations, whereas the dashed purple lines show inferred *Sargassum* transport not observed in the satellite record. Basemaps from Climate Data Toolbox^51^.

**Extended Data Fig. 3:**
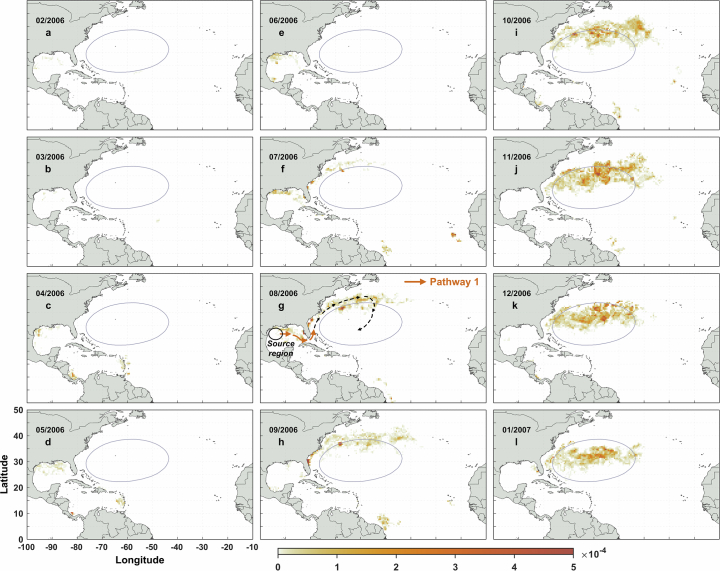
A case illustrating the typical transport pathway of *Sargassum* from the Gulf of Mexico to the Sargasso Sea and adjacent waters for the pre- Great Atlantic *Sargassum* Belt era (2000–2010). The color scale indicates mean fractional coverage (1×10^−4^ = 0.01%), and the relevant *Sargassum* transport pathway is shown in panel g. Panels identified by month from February 2006 (**a**) until January 2007 (**l**). Basemaps from Climate Data Toolbox^51^.

Instead, such a dramatic decline appears related to changes in the historical primary source region for the SS—the northwestern GoM^32,33^. Analysis of *Sargassum* distribution maps (Extended Data Figs. 1 and 2 and Supplementary Movie 1) along with ocean surface currents and winds indicated that before 2015 *Sargassum* first appeared in the northwestern GoM around February, then expanded eastward through Gulf currents and eddies (Pathway 1 in Fig. 4b)^32,33^. *Sargassum* then moved into the Gulf Stream Region (GSR) via the Loop and Florida Currents during the spring/summer (Fig. 4a,b). Subsequent southward movement within the SS followed local currents and wind convergence zones during fall/winter (Supplementary Figs. 2–4). Ocean currents and winds play key roles in regulating *Sargassum* transport and distribution^19,33–35^, as shown in Extended Data Fig. 3. The historic transport cycle closed with *Sargassum* passing into the western Caribbean via the Windward and Mona Passages and returning to seed the GoM via the Caribbean and Yucatan Currents (Fig. 4b).

Years 2011–2014 represent a transition period, during which the SS received *Sargassum* from both the northwestern GoM (Pathway 1) and the GASB (Pathways 2 and 3; Fig. 4c). For the latter, *Sargassum* was first transported from the tropical Atlantic to the eastern Caribbean via the North Equatorial Current, arriving in early summer. From here, a portion of the *Sargassum* could be directly transported northwestward to the SSS by the Antilles Current (Pathway 3 in Fig. 4c). Other GASB-sourced *Sargassum* arriving in the CS was transported to the GoM via the Caribbean and Yucatan Currents during August–December (Pathway 2). Mirroring Pathway 1, a portion of this *Sargassum* was subsequently transported to the GSR via the Loop Current, Florida Current and Gulf Stream.

**Extended Data Fig. 4:**
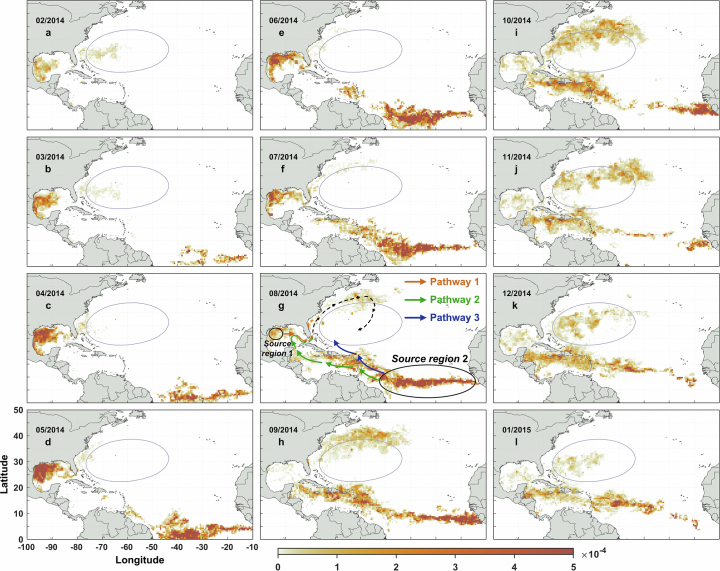
A case illustrating the typical transport pathway of *Sargassum* from the Gulf of Mexico and the tropical Atlantic to the Sargasso Sea and adjacent waters for the early Great Atlantic *Sargassum* Belt period (2011–2014). The color scale indicates mean fractional coverage (1×10^−4^ = 0.01%), and the relevant *Sargassum* transport pathways are shown in panel g. Panels identified by month from February 2014 (**a**) until January 2015 (**l**). Basemaps from Climate Data Toolbox^51^.

During this transition period (Fig. 4c), the northwestern GoM continued to have a greater impact on delivery of *Sargassum* to the NSS (Pathway 1) than the GASB (Pathway 2). For example, during February–July 2014 (Extended Data Fig. 4a–f), *Sargassum* grew in the northwestern GoM and later supplied the GSR, mirroring the 2000–2010 pattern (Pathway 1). There was almost no *Sargassum* in the CS, so GoM *Sargassum* biomass must have been locally seeded. By July 2014 (Extended Data Fig. 4f), substantial *Sargassum* had already accumulated in the GSR, a precursor for formation of the fall/winter NSS peak observed in the pre-GASB era (compare the North Atlantic *Sargassum* footprint in Extended Data Fig. 3f,g to the same region and times in Extended Data Fig. 4).

**Extended Data Fig. 5:**
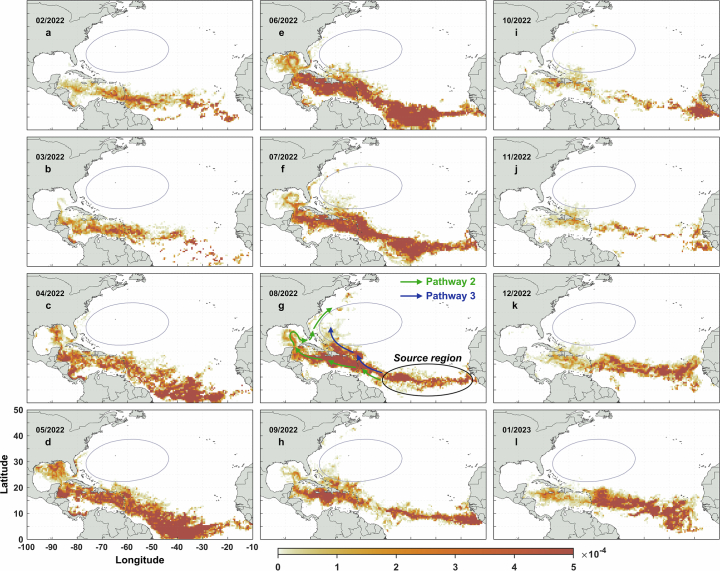
A case illustrating the typical transport pathway of *Sargassum* from the tropical Atlantic to the Sargasso Sea and adjacent waters for the recent Great Atlantic *Sargassum* Belt era. (2015–2023, excluding calendar years of 2019 and 2021). The color scale indicates mean fractional coverage (1×10^−4^ = 0.01%), and the relevant *Sargassum* transport pathways are shown in panel g. Panels identified by month from February 2022 (**a**) until January 2023 (**l**). Basemaps from Climate Data Toolbox^51^.

During the recent GASB era, a new scenario of *Sargassum* transport to the SS was observed (Fig. 4d), which manifested in the significant NSS *Sargassum* decline relative to preceding time periods. By Pathway 2, the GASB supplied substantial *Sargassum* to the GoM starting in March and continuing through August (Extended Data Fig. 5b–g). Of this, a small amount was carried by the Gulf Stream to just north of the NSS (Extended Data Fig. 5f,g). Meanwhile, *Sargassum* was also transported directly from the GASB to the SSS via Pathway 3 (Extended Data Fig. 5e–h), with a very small portion reaching the NSS (Extended Data Fig. 5g,h).

## Ecological underpinnings of the *Sargassum* decline in the NSS

*Sargassum* has approximately a 1-year lifespan^32^ and is well understood to naturally grow and decline over time^5^ in stages marked by changes in algal coloration, buoyancy and accumulation of diverse epibionts^36–40^. These changes collectively result in *Sargassum* clumps sinking. Under adverse or marginal environmental conditions, *Sargassum* decline can accelerate to the point of sinking in a matter of days^36,37^. Morphotypes, determined from in situ observations, differ in terms of their structure, ecological value^10^ and response to seawater characteristics^36,37,41,42^.

Temperature strongly influences *Sargassum* growth rates, with laboratory and field experiments demonstrating positive impacts under moderate conditions and negative impacts at both extreme thresholds^36,37,41,42^. Optimal growth and health occur between ~23 and 28 °C, with peak growth rates at different temperatures for each morphotype and *Sn_n*/*Sf* generally faster growing than *Sn_w*^37,43^. Within this range, increases in temperature enhance growth. At lower temperatures (<20 °C), growth slows (*Sf*/*Sn_n*) or halts (*Sn_w*)^37,43^. All *Sargassum* declines in both growth and health (for example, loss of blades and floats, darkening colour, tissue decay) at temperatures approaching and above 30 °C, with *Sn_n* least tolerant starting at 28 °C (refs. ^37,41–43^). Nutrients also influence *Sargassum* growth and productivity^44,45^; however, there is no evidence yet of differing morphotype responses to nutrient conditions.

The lack of *Sargassum* in the northwestern GoM source region from 2015 through 2023 (Fig. 2d) may indicate cessation of Pathway 1, resulting in NSS *Sargassum* decline due to a lack of biomass arriving from the GoM. When GASB-sourced *Sargassum* reaches the GoM, its massive abundance may overwhelm the existing local population and compete for available nutrients. Furthermore, warming trends in the GoM, particularly since 2013 and amplified in the western sector, have pushed year-round background temperatures towards algal thermal thresholds with continued, accumulating physiological impacts^46,47^. Superimposed on this warming, marine heatwaves in the GoM have sharply increased in duration, frequency and intensity since 2015^46^. Short but discrete heatwave events may be enough to suppress the local *Sargassum* population in the northwestern GoM to a point from which it has not been able to rebound and may also impact *Sargassum* arriving from the GASB.

Along Pathways 2 and 3 through the tropical Atlantic and CS, *Sargassum* experiences water temperatures of 28° to >31 °C during much of the year, with negative effects that contribute to interannual fluctuations in algal health. For example, *Sn_w* in the GASB was healthy in December 2014 but in decline one year later in December 2015^48^. Biomass from the GASB may thus already be senescent by the time it reaches the GoM or NSS, as Pathway 2 is nearly twice as long as Pathway 1 and/or the *Sargassum* encountered unfavourable environmental conditions while in transit.

**Extended Data Fig. 6:**
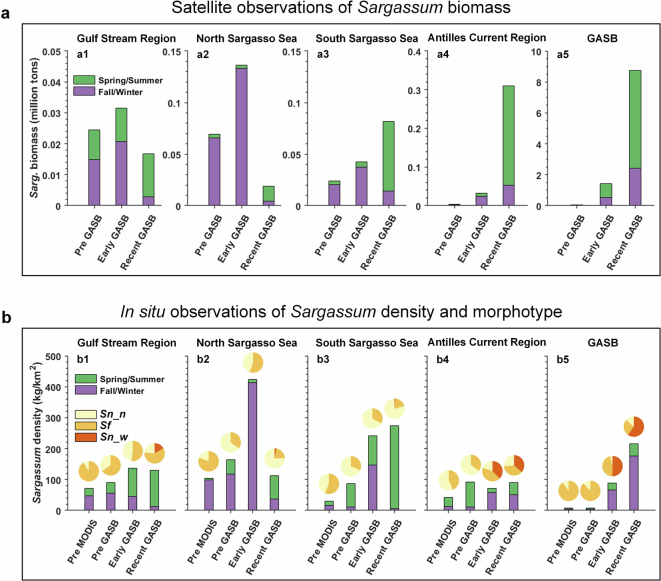
(A) Satellite-observed regional mean *Sargassum* biomass across three time periods. (B) *In situ* regional weighted mean *Sargassum* densities and morphotypes across four time periods. Pie charts in **b** show relative abundance of each morphotype as proportions of total *Sargassum* density. *Sn_n*, *Sf*, and *Sn_w* represent *Sargassum natans* var. *natans, fluitans*, and *natans* var. *wingei*, respectively, while GASB and MODIS abbreviate Great Atlantic *Sargassum* Belt and Moderate Resolution Imaging Spectroradiometer, respectively. The pre-MODIS, pre-GASB, early GASB, and recent GASB timeframes correspond to 1993–1999, 2000–2010, 2011–2014, and 2015–2023, respectively. Sub-panels identified by region (1, Gulf Stream Region; 2, North Sargasso Sea; 3, South Sargasso Sea; 4, Antilles Current Region; 5, Great Atlantic *Sargassum* Belt Region).

Interestingly, GASB-sourced *Sargassum*, often *Sn_w*, transported through the GoM and along the GSR via Pathway 2 (Extended Data Figs. 1d3–f3 and 2a3) has not populated the NSS (Fig. 2eiii,iv)^9^. GASB-sourced *Sargassum* arrives to the area north of the NSS in the same summer months as that preceding NSS peak abundance during the pre-GASB era (Extended Data Fig. 1e1,e3,f1,f3). Any *Sn_w* carried by the Gulf Stream towards the NSS via Pathway 2 encounters cold (18° to 22.5 °C; Supplementary Fig. 1), oligotrophic waters and does not thrive; by October, when the *Sargassum* footprint shifts south into the NSS, no *Sn_w* remains alive to be introduced to the SS (Extended Data Fig. 2b3). *Sn_n* and *Sf* persist in the SS in ecologically relevant abundances, albeit at lower levels than historically (Fig. 2e and Extended Data Fig. 6b) that may be below satellite detection limits^48^. Additionally, *Sargassum* transported via Pathway 3 has been unable to establish or bloom in the SSS, potentially due to poor physical condition on arrival or limited nutrient supply given the region’s shallow MLDs (Supplementary Fig. 1) and lack of major riverine inputs.

## What happened in the anomalous years of 2019 and 2021

**Extended Data Fig. 7:**
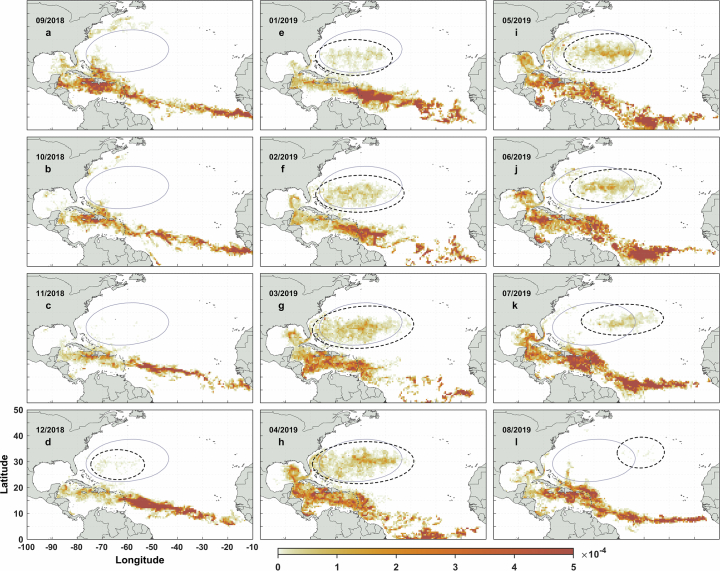
Formation and evolution of an anomalous *Sargassum* bloom occurred in the Sargasso Sea and its surrounding waters during 2019. The *Sargassum* patterns associated with this bloom are highlighted with dashed black ellipses, and the color scale indicates mean fractional coverage (1×10^−4^ = 0.01%). The time period shown in this figure was selected to highlight bloom initiation and termination, and does not correspond to the months removed from Fig. 1 (that is, 2019 calendar year). Panels identified by month from September 2018 (**a**) until August 2019 (**l**). Basemaps from Climate Data Toolbox^51^.

**Extended Data Fig. 8:**
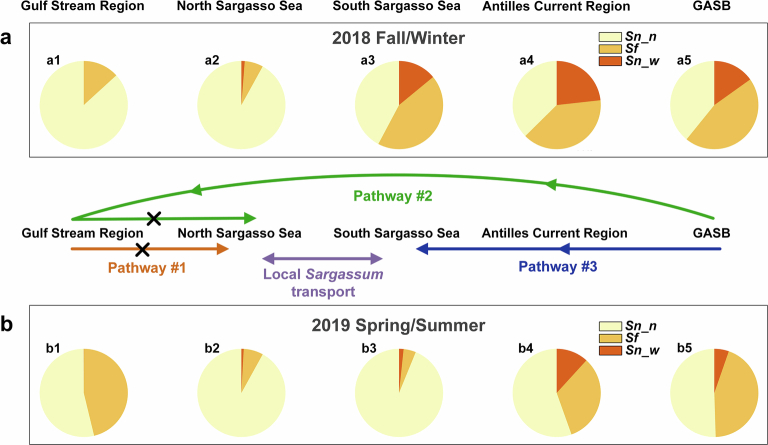
In situ observations of *Sargassum* morphotype proportions during (A) 2018 fall/winter and (B) 2019 spring/summer in five regions. *Sn_n*, *Sf*, and *Sn_w* represent *Sargassum natans* var. *natans, fluitans*, and *natans* var. *wingei*, respectively, while GASB abbreviates Great Atlantic *Sargassum* Belt. *Sargassum* transport pathways connecting different regions are indicated by solid lines with arrows. Sub-panels identified by region (1, Gulf Stream Region; 2, North Sargasso Sea; 3, South Sargasso Sea; 4, Antilles Current Region; 5, Great Atlantic *Sargassum* Belt Region).

Two anomalous years were excluded from descriptions of *Sargassum* long-term distribution patterns and transport pathways, but they were included in satellite data time-series analysis and statistical tests of the temporal declines. In 2019, the GASB supplied *Sargassum* to the northern edge of the NSS through Pathway 2 and to the SSS via Pathway 3 (Extended Data Fig. 7a). As with other recent GASB years, Pathway 2 did not seed the NSS and there was no obvious southward transport of *Sargassum* within the SS (Extended Data Fig. 7b,c). Moreover, despite parallels between morphotype composition in the GSR and NSS during fall/winter of 2018 (Extended Data Fig. 8a1,a2), the bloom that developed in January 2019 was offset by many months. *Sargassum* from Pathway 3 also did not appear to supply the 2019 bloom, as biomass in the SSS at the time of bloom initiation was disconnected from the GASB (November–December 2018; Extended Data Fig. 7c,d). Whereas proportional distribution of *Sargassum* morphotypes sampled during late 2018 indicates strong similarity between the GASB, Antilles Current Region (ACR) and SSS (Pathway 3; Extended Data Fig. 8a3–a5), these similarities did not persist into the spring/summer of 2019 (Extended Data Fig. 8b3–b5). Thus, *Sargassum* from the local standing population of *Sn_n* bloomed during 2019 in the SSS—an area where such formation was not previously observed. Notably, local conditions were probably favourable for *Sargassum* growth, evidenced by deeper MLD and warmer-than-usual water temperatures (Supplementary Figs. 5c2 and 6a2–a9). By spring/summer 2019, the NSS and SSS showed roughly equivalent morphotype densities (Extended Data Fig. 8b2,b3), which were distinct from either upstream region. Once established, the bloom’s eastward transport was facilitated by winds and surface currents (detailed in Supplementary Text 1).

**Extended Data Fig. 9:**
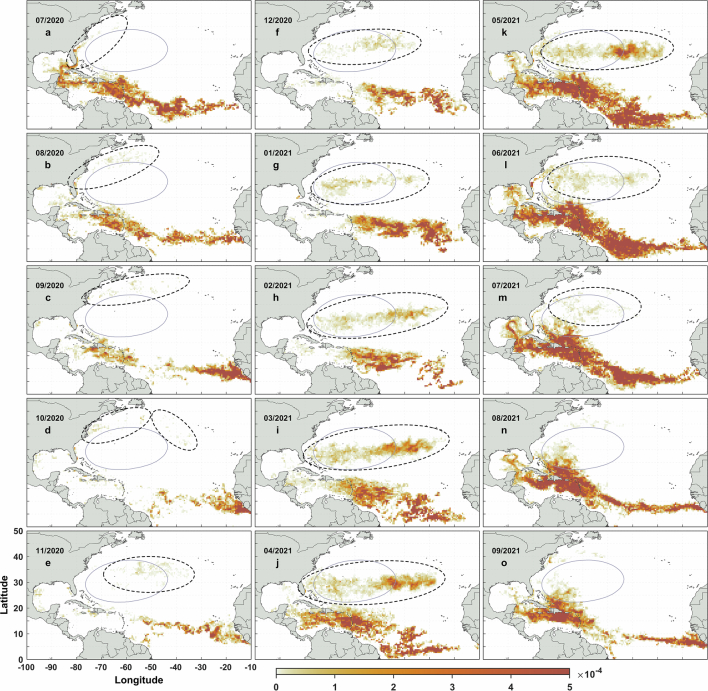
Formation and evolution of an anomalous *Sargassum* bloom occurred in the Sargasso Sea and its surrounding waters during 2021. The *Sargassum* patterns associated with this bloom are highlighted with dashed black ellipses and the color scale indicates mean fractional coverage (1×10^−4^ = 0.01%). The time period shown in this figure was selected to highlight bloom initiation and termination, and does not correspond to the months removed from Fig. 1 (that is, 2021 calendar year). Panels identified by month from July 2020 (**a**) until September 2021 (**o**). Basemaps from Climate Data Toolbox^51^.

**Extended Data Fig. 10:**
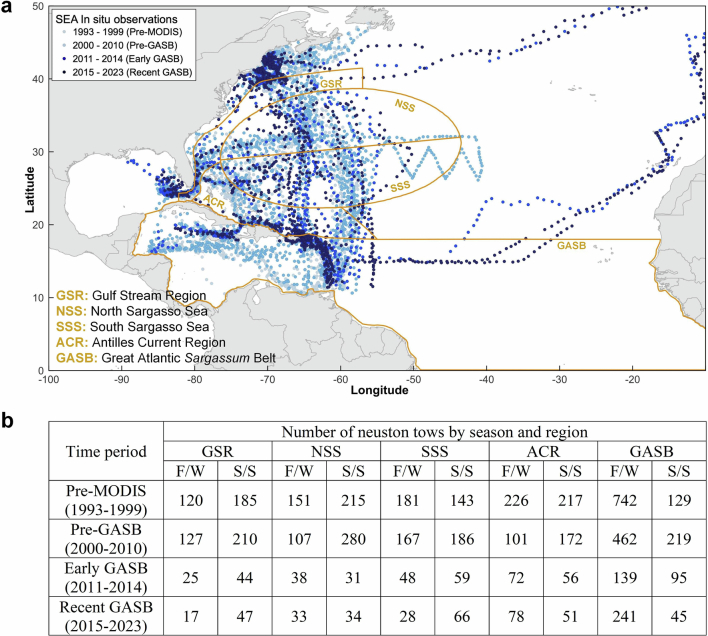
In situ *Sargassum* observations. (**a**) Locations of surface neuston net tows in the North Atlantic Ocean during four periods, with (**b**) showing the number of samples collected in each region and time period. Each sample provides *Sargassum* morphotype and density data. Seasons divided into fall/winter (F/W) and spring/summer (S/S). MODIS and SEA abbreviate Moderate Resolution Imaging Spectroradiometer and Sea Education Association, respectively. Basemaps from Climate Data Toolbox^51^.

For the 2021 event, the GASB supplied *Sargassum* to the NSS and surrounding waters through Pathway 2 (Extended Data Fig. 9a,b), albeit pushed east beyond the SS boundary (Extended Data Fig. 9d). Subsequently, the *Sargassum* travelled southward (as normal) while expanding both eastward and westward under the influence of winds and surface currents (Supplementary Fig. 7). Similar to the 2019 event, sustained warmer water temperatures and anomalously deep MLDs (Supplementary Figs. 6b1–b10 and 7c5) probably supported algal growth (detailed in Supplementary Text 2). Unfortunately, in situ surveys in late 2020 did not sample the bloom precursor, and no surveys were conducted in the NSS, SSS or ACR during spring/summer 2021 (Extended Data Fig. 10).

## The future of *Sargassum* in the Sargasso Sea

Associated with emergence and expansion of the GASB, basin-scale transport pathways and *Sargassum* morphotype composition shifted, garnering extensive attention from various stakeholders. This study describes NSS changes and puts forth a hypothesis that links two contributing processes. First, the northwestern GoM, the historic source of fall/winter *Sargassum* abundance peaks in the NSS (Pathway 1), has shown minimal biomass during spring in the recent GASB era (Fig. 2d). Increasing GoM temperatures, marine heatwave events and nutrient competition may have reduced and/or negatively impacted growth potential of the locally sourced population.

Second, although abundant in the now-persistent GASB, *Sn_w* transported north in the GSR (via Pathway 2) never established or bloomed in the NSS, possibly due to the slow growth rate of this morphotype, thermal tolerances, timing of arrival and/or limited nutrient supply. Furthermore, longer travel times and distances, in addition to high temperature conditions in the tropics, may impact the ecological health of all GASB-sourced morphotypes as they travel throughout the basin before reaching the NSS. As a result of these processes, the historically consistent fall/winter NSS biomass peak^12,13^ is diminished during the recent GASB era, making spring/summer biomass that mirrors GASB seasonality more pronounced (Fig. 3c and Extended Data Fig. 6a2–a5).

Together, we interpret these changes to represent an ongoing regime shift in holopelagic Atlantic *Sargassum*. The environmental changes driving this regime shift probably include specific events^18–20,34^ (for example, the 2010 North Atlantic Oscillation anomaly that may have formed the GASB) and broader alterations in habitat suitability (for example, warming SSTs) in historical source regions^46,47^. The question is whether such dramatic changes reported in this study will continue in the future. Since its first occurrence in 2011, the GASB has shown no sign of decline, but instead has intensified in recent years. This intensification appears to have become a new norm because the tropical Atlantic provides more favourable conditions (light, temperature, nutrients) than the SS to maintain a large population of *Sargassum*. If our hypothesis regarding *Sargassum* declines in both the NSS and the historic northwestern GoM source region is true, the observed regime shift in *Sargassum* distributions and seasonality should also be expected to continue. We expect to test this hypothesis as additional data on *Sargassum* abundance, distribution, environmental tolerances and growth dynamics become available.

## Methods

### In situ *Sargassum* data collection and analysis

Twice daily (~noon and midnight local time) neuston net (1.0 m × 0.5 m frame, 333-um mesh) tows were conducted during annually repeated cruises (1993–2023) through the North Atlantic aboard Sea Education Association (www.sea.edu) vessels the *SSVs Westward* and *Corwith Cramer*. In most years, the vessels followed an annual cycle departing Massachusetts southbound in October, sailing in the western tropical Atlantic and Caribbean from November through April and transiting north through the Sargasso Sea (SS) in May. In total, 5,587 neuston tows were collected across the five studied regions and four time periods (Extended Data Fig. 10).

For each collection, the neuston net was deployed from a boom extended (5 m) off the port side of the ship while sailing on a port tack. Consequently, when present, the windrows of *Sargassum* were crossed perpendicularly and any bow wake effect was minimized. Tow speed was maintained at two knots for a duration of 30 minutes for a typical tow distance of approximately 1.0 nm. Tow distance (m) was calculated in earlier years (1993–2003) as the difference between recorded GPS locations at the start and end of each tow and in later years (2004–2023) from minute-by-minute GPS locations recorded during each tow.

Standard net processing of collected *Sargassum* (and other macroalgae) included rinsing with seawater to remove entangled zooplankton biomass, meroplankton larvae, microplastics and tar. *Sargassum* specimens were sorted by species and morphotype^5,9^, patted dry with paper towels and weighed using a spring scale to yield *Sargassum* mass (g) for each morphotype.

Analysis of neuston tow data were organized by ecological year (March–February) and season. An ecological year corresponds with the start of the annual spring bloom period, which is March for the SS region. Therefore, January and February were grouped with the prior year. Seasons were designated as follows: spring (March–May), summer (June–August), fall (September–November) and winter (December–February). Weighted arithmetic mean *Sargassum* density was calculated from the sum of *Sargassum* mass divided by the sum of tow area for all tows in a given year (or time period) for a given region. Densities were scaled to kg km^−^^2^ for easier comparison with remote sensing data. In most years, neuston tow collections occurred during both the spring/summer and fall/winter seasons in a region. However, for a given region, one of the sampling seasons might have been missed due to a change in cruise track. For example, from fall 2014 through spring 2017, the vessel followed an annual cycle that included a northern trans-Atlantic crossing from Massachusetts to the Mediterranean (June to October), a southern trans-Atlantic track arriving in the Caribbean in mid-December, winter sailing in the Caribbean and the northbound transit through the SS in May. Thus, the traditional fall/winter sampling periods in the North Sargasso Sea (NSS) and South Sargasso Sea (SSS) were missing during these years.

### Satellite-based *Sargassum* data products

Mapping and quantification of pelagic *Sargassum* using satellite observations for the period March 2000 to February 2024 (inclusive) were conducted as described in ref. ^52^. Briefly, Level-1 satellite data from Moderate Resolution Imaging Spectroradiometer (MODIS) on Aqua and Terra collected within the geographic range 20° S–50° N, 98° W–15° E were acquired from NASA archives (https://oceancolor.gsfc.nasa.gov). Data were processed with partial atmospheric correction to derive Rayleigh-corrected reflectance (Rrc) as described in ref. ^53^, from which the Alternate Floating Algae Index (AFAI) was computed as:

$$\,\begin{array}{c}\mathrm{AFAI}=\mathrm{Rrc}(748)-[\mathrm{Rrc}(667)+(\mathrm{Rrc}(869)\\ -\mathrm{Rrc}(667))\times (748-667)/(869-667)]\end{array}$$

This AFAI product and Rrc from seven MODIS wavebands (412, 443, 488, 547, 678, 748 and 869) were mapped to a cylindrical equidistant projection at 1-km resolution. Data were then linearly scaled (8-bit) within the range −0.001 to 0.003 for AFAI and logarithmically scaled within 0.0075–0.2 for Rrc. Pixels identified as land or cloud (defined using the Level-2 processing flags ‘LAND’ and ‘CLDICE’, respectively) were removed from analysis, as were any pixels within 10 km of LAND or 2 km of CLDICE.

The data were then analysed in 256 × 256 pixel subregions using the Res-UNet model developed in ref. ^52^, through which *Sargassum*-containing pixels were identified. For each contiguous patch of *Sargassum*-containing pixels (excluding any patches smaller than 3 pixels), ΔAFAI of each *Sargassum*-containing pixel was calculated as its AFAI minus the median AFAI for all non-*Sargassum* pixels within a 13-pixel dilation. From this, ΔAFAI was used to estimate the pixel’s *Sargassum* fractional density by linearly scaling to 4.41 × 10^−2^ (corresponding to 100% sub-pixel fractional density)^31^. Monthly and annual composites of *Sargassum* areal density were calculated as the mean fractional density within each calendar month (year) for 0.5° × 0.5° grid. A mean conversion factor of 3.34 kg wet biomass m^−^^2^ (ref. ^54^) was further used to convert areal coverage to biomass. Notably, differentiation between *Sargassum* morphotypes was not attempted in this study, as the spectral resolution of MODIS is insufficient for such determinations. These *Sargassum* data products were distributed through the *Sargassum* Watch System (SaWS, https://optics.marine.usf.edu/projects/saws.html) by the Optical Oceanography Laboratory at the University of South Florida, which have been used widely by the research and management communities.

### Statistical analyses

Visual analysis of the *Sargassum* density maps (2000–2023) showed high interannual variability. Among this variability, we identified three time periods sharing commonalities in *Sargassum* footprint and connectivity pathways (Fig. 1). The first transition between these time periods corresponded to the initial formation of the GASB in 2011, whereas the second coincided with a lack of GoM-sourced *Sargassum* reaching the NSS (starting in 2015). For the satellite-derived NSS *Sargassum* abundance time series, we performed two-sample Student’s *t*-tests to evaluate the null hypothesis that the annual *Sargassum* biomass in the various time periods were from independent data distributions with the same means. Before performing the *t*-tests, two-sample *F*-tests were performed to determine if the variances of these two populations were equal. If so (at α = 0.05), the *t*-test was performed without assuming homoscedasticity. These analyses were performed in MATLAB version 2017a. Notably, these statistical tests were performed for satellite data only, as the weighting approach for in situ data (as described above) precludes such analysis for these time series.

### Environmental data products

To understand the mechanisms influencing the spatial and temporal changes of *Sargassum* in the SS and adjacent waters, we analysed various datasets to examine the changes in environmental conditions, including SST, Chlorophyll-a concentration, winds, MLD and surface currents. The details of these datasets are summarized in Supplementary Table 1.

## Online content

Any methods, additional references, Nature Portfolio reporting summaries, source data, extended data, supplementary information, acknowledgements, peer review information; details of author contributions and competing interests; and statements of data and code availability are available at 10.1038/s41561-025-01863-5.

## Supplementary information

Supplementary Information Supplementary Texts 1–3, Figs. 1–9 and Table 1 and caption for Supplementary Movie 1.

Supplementary Video 1 Animation of the monthly distribution of mean areal density of *Sargassum* in the Gulf of Mexico, Caribbean Sea and the North Atlantic Ocean during March 2000 to February 2024.

## Data Availability

*Sargassum* wet biomass data derived from MODIS satellite observations spanning March 2000 to February 2024 for all subregions defined in this work are available at 10.17632/zcyd5wvncc.1 (ref. ^55^), whereas weekly *Sargassum* density maps can be accessed through the *Sargassum* Watch System (SaWS) at https://optics.marine.usf.edu/projects/SaWS.html. In situ measurements of *Sargassum* density and morphotypes are available at 10.17632/jwj426c28b.1 (ref. ^56^). All environmental datasets used in this study, along with links to their respective data sources, are summarized in Supplementary Table 1.

## Code availability

The MATLAB code used for data analysis and figure preparation is available from the corresponding author upon request.
