# Supplementary material for: Dramatic decline of Sargassum in the north Sargasso Sea since 2015
Source: Nat Geosci. 2025 Dec 4;18(12):1266–72. doi: 10.1038/s41561-025-01863-5 (PMC12685743; doi:10.1038/s41561-025-01863-5)
Supplement: Supplementary file 1 — Supplementary Texts 1–3, Figs. 1–9 and Table 1 and caption for Supplementary Movie 1. [file 41561_2025_1863_MOESM1_ESM.pdf]

nature geoscience

Supplementary information https://doi.org/10.1038/s41561-025-01863-5

Dramatic decline of *Sargassum* in the north Sargasso Sea since 2015

In the format provided by the authors and unedited

This File Includes:

Supplementary Texts 2
Supplementary Text 1 2
Supplementary Text 2 3
Supplementary Text 3 3
Supplementary Figures 5
Supplementary Table 14
Supplementary Video 1 Caption 15

Other Supplementary Information for this manuscript includes the following:

Supplementary Video 1

Supplementary Texts

Supplementary Text 1

Anomalous *Sargassum* bloom event in the Sargasso Sea in 2019

The development and evolution of the anomalous *Sargassum* bloom that occurred in the Sargasso Sea (SS) and adjacent waters during 2019 are illustrated in the sequential maps of monthly *Sargassum* areal density, as shown in **Extended Data Fig. 7**. Initially, the SS was nearly *Sargassum* free in November 2018 (**Extended Data Fig. 7c**), however, large amounts of *Sargassum* began to appear in December 2018 (**Extended Data Fig. 7d**). From December 2018 to April 2019, the majority of *Sargassum* in the northwestern Atlantic was concentrated within the SS (**Extended Data Figs. 7d–h**). However, by May 2019 (**Extended Data Fig. 7i**), a distinct separation occurred, with *Sargassum* in the region splitting into two branches: one following the path of the Gulf Stream, while the other followed the eastward/southeastward surface currents (**Supplementary Figs. 5b7–9**), subsequently migrating eastward (**Extended Data Figs. 7i–k**). By August 2019, these unusual *Sargassum* patterns had essentially dissipated (**Extended Data Fig. 7l**). Notably, this timing is distinct from the traditional timing for SS blooms, whereby a normal ecological year spans from March to February. As such, the 2019 calendar year was used when excluding this bloom (and the 2021 bloom) from the composite in **Fig. 1c**.

To understand the mechanisms underlying this anomalous *Sargassum* bloom, we analyzed environmental conditions including winds, surface currents, mixed layer depth (MLD), and sea surface temperature (SST), with results shown in **Supplementary Figs. 5,6**. During October and November 2018, little or no *Sargassum* was observed in the SS (**Extended Data Figs. 7b–c**). A clear *Sargassum* pattern emerged in the SS in December 2018 (**Extended Data Fig. 7d**), a time period in which growth-promoting conditions were present, characterized by deeper MLDs in the region of 20°N–30°N and 75°W–50°W and warmer-than-usual water temperatures (**Supplementary Figs. 5c1–2,6a2**). These conditions, coupled with eastward transport driven by stronger-than-usual eastward winds around 30°N (**Supplementary Fig. 5a2**) and intensified eastward surface currents (28°N–32°N and 75°W–55°W; **Supplementary Fig. 5b2**), led to both increased *Sargassum* abundance and its eastward movement between December 2018 and January 2019 (**Extended Data Figs. 7d–e**). While deeper MLDs and warmer temperatures do not generally co-occur, the anomaly values reported here were calculated against a climatological baseline spanning 2002–2023. Due to global warming trends, 'cooler' waters associated with deeper MLDs may still be anomalously warm relative to historical values.

The combination of growth-promoting conditions and transport mechanisms continued to influence *Sargassum* distribution and abundance in subsequent months. For instance, during February 2019, deeper MLDs east of 60°W and consistently warmer-than-usual waters (**Supplementary Figs. 5c4,6a4**) supported *Sargassum* growth, while eastward winds near 30°N (50°W–40°W; **Supplementary Fig. 5a4**) and related southeastward surface currents (28°N–32°N and 50°W–40°W; **Supplementary Fig. 5b4**) facilitated its transport from 45°W to 40°W (**Extended Data Figs. 7f–g**). Similarly, the continuous eastward transport of *Sargassum* during May–July 2019 (**Extended Data Figs. 7i–k**) appears to be driven by eastward/southeastward surface currents with accompanying eastward winds (**Supplementary Figs. 5b7–9,5a7**). Notably, the anomalously warm waters observed throughout this event provided favorable conditions for *Sargassum* growth (**Supplementary Figs. 6a2–9**), as these floating macroalgae typically exhibit enhanced physiological performance and growth rates in warm waters.

To understand the origins of this unusual *Sargassum* event, we analyzed satellite observations. The *Sargassum* areal density map from September 2018 (**Extended Data Fig. 7a**) revealed two key patterns: a scarcity of *Sargassum* in the northwestern Gulf of Mexico, and notable populations in the Gulf Stream Region

(GSR) and the southwestern SS. These distribution patterns align with *Sargassum* transport Pathways #2 and #3 respectively (**Fig. 4**), both originating from the Great Atlantic *Sargassum* Belt (GASB). However, this did not result in the establishment of a bloom (**Extended Data Figs. 7b–c**). Instead, a bloom of locally supplied *Sn_n* develops in the SS beginning in January 2019 (**Extended Data Figs. 7e–k**).

Supplementary Text 2

<u>Anomalous *Sargassum* bloom event in the Sargasso Sea in 2021</u>

Another anomalous *Sargassum* bloom occurred in 2021, with its entire evolution documented in **Extended Data Fig. 9**. During July–November 2020 (**Extended Data Figs. 9a–e**), *Sargassum* was notably absent along Pathways #1 and #3. However, distinct *Sargassum* transport was observed along Pathway #2 (**Extended Data Fig. 9a**), indicating its origin in the GASB.

In October, the potential beginnings of a *Sargassum* bloom were observed far east of the historical footprint (**Extended Data Fig. 9d**). The bloom was initially concentrated between 32°N–40°N and 55°W–35°W in November (**Extended Data Fig. 9e**), then *Sargassum* was transported westward by December 2020 (**Extended Data Fig. 9f**), driven by westward winds and surface currents (**Supplementary Figs. 7a2,b2**). During this period, favorable growth conditions, including warmer temperatures and deeper MLDs (**Supplementary Figs. 6b2,7c2**), likely promoted *Sargassum* growth.

From January to March 2021, *Sargassum* was transported even farther eastward (**Extended Data Figs. 9g–i**) by eastward winds and southeastward surface currents (**Supplementary Figs. 7a4–5,b4–5**). *Sargassum* density increased largely east of 50°W between February and March 2021 (**Extended Data Figs. 9h–i**), supported by favorable growth conditions including warmer water temperatures and deeper MLDs (**Supplementary Figs. 6b4–6,7c5**). Between March and May 2021, these distinct *Sargassum* patterns persisted between 25°N–35°N and 50°W–30°W (**Extended Data Figs. 9i–k**), likely maintained by consistently warm water temperatures (**Supplementary Figs. 6b6–8**).

The subsequent decline in *Sargassum* abundance and coverage from May to August 2021 (**Extended Data Figs. 9k–n**), coincided with less favorable growth conditions, particularly shallower MLDs (**Supplementary Figs. 7c8–10**). This anomalous bloom was remarkable for reaching eastern extents (24°W–27°W) between February and May 2021 (**Extended Data Figs. 9h–k**) not frequently seen in the satellite record (**Figs. 1a,b**). While intensified eastward winds and surface currents (**Supplementary Figs. 7a4,a7,b4,b7**) enabled this expansion, consistently warmer-than-usual water temperatures (**Supplementary Figs. 6b1–10**) supported the bloom's persistence by providing favorable conditions for *Sargassum* growth throughout the event. It should be noted that due to changes in cruise plans, we lack *in situ* observations of *Sargassum* morphotype for this anomalous event, preventing analysis of dominant species and regional connectivity.

Supplementary Text 3

<u>Satellite and *in situ* observations of *Sargassum* changes in the North Atlantic Ocean</u>

Satellite observations have revealed substantial changes in *Sargassum* biomass not only in the North Sargasso Sea (NSS) and the GASB (**Figs. 1,2,3; Extended Data Fig. 6**), but also in adjacent regions including

the GSR, South Sargasso Sea (SSS), and Antilles Current Region (ACR). During the recent GASB era (2015–2023), the GSR showed a decreasing trend in *Sargassum* abundance, with *Sargassum* peak season shifting from fall/winter to spring/summer (**Extended Data Fig. 6a1**). In contrast, both the SSS and ACR have experienced increases in *Sargassum* biomass during this period, mirroring changes observed in the GASB, with peak abundances occurring in spring/summer (**Extended Data Figs. 6a3–4**).

In situ observations of *Sargassum* density and peak seasons (**Extended Data Fig. 6b**) largely corroborate satellite observations in the GSR, NSS and SSS. However, a notable discrepancy exists in the ACR and GASB, where *in situ* observations indicate peak abundance in fall/winter, contrary to the spring/summer peaks observed by satellite during the recent GASB era (2015–2023). This discrepancy may be attributed to sampling bias in data collection. For example, during the 2015–2023 period, 241 neuston tows were conducted in fall/winter in the GASB compared to only 45 in spring/summer (**Extended Data Fig. 10b**). Detailed annual and seasonal variations in *Sargassum* abundance/density for each region are further documented in **Supplementary Figs. 8,9** for satellite and *in situ* observations, respectively.

Supplementary Figures

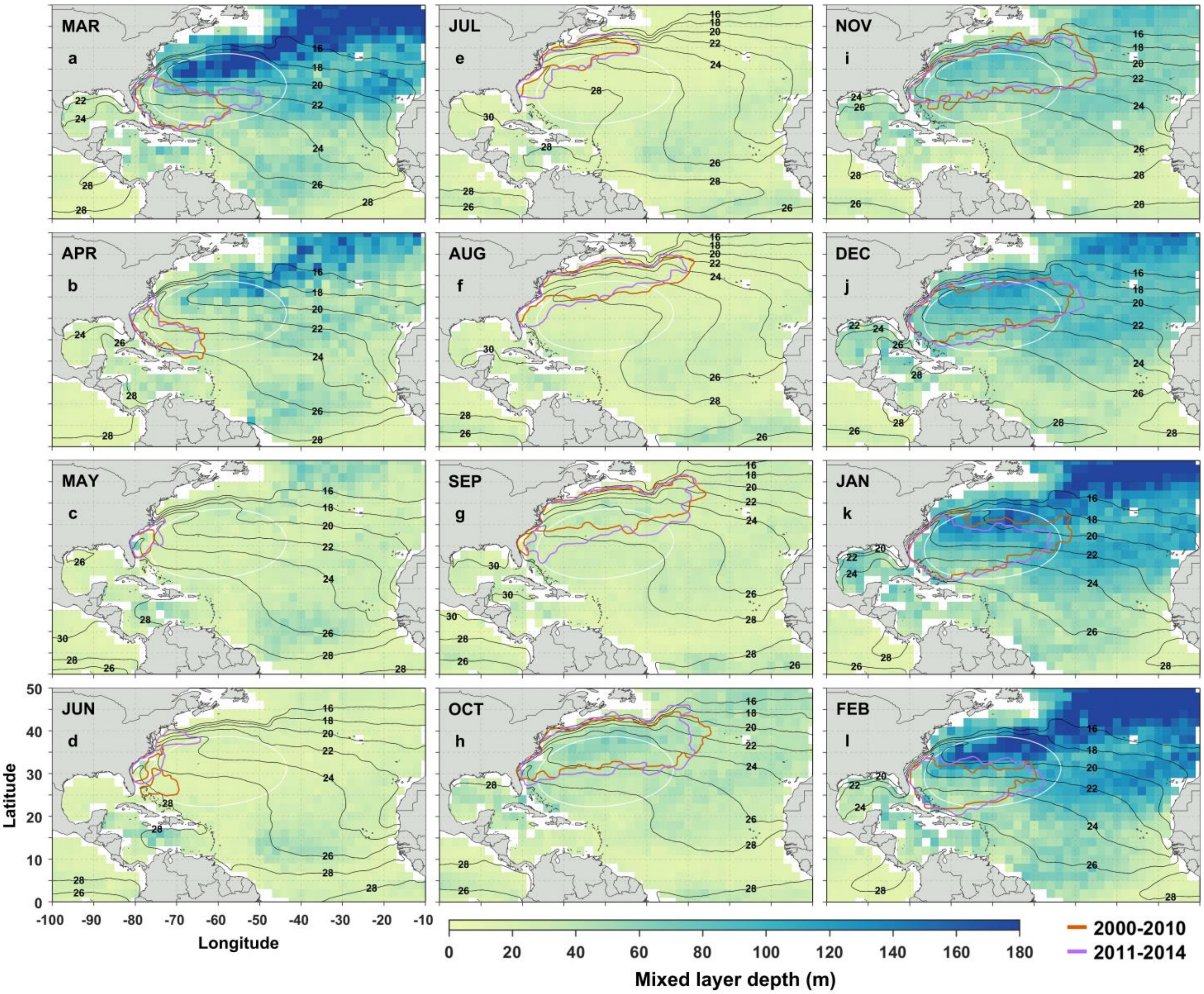

Supplementary Fig. 1 | Monthly climatological distributions of MLD, measured by Argo floats in the Gulf of Mexico, Caribbean Sea, and North Atlantic between 2001 and 2022. White color means no data. The solid orange and purple curves in each subplot represent the *Sargassum* boundaries in the SS and its surrounding waters for the pre-GASB era (2000–2010) and early GASB period (2011–2014), respectively. Solid black lines in each subplot indicate SST contours, derived from monthly climatological maps of MODIS/Aqua observations during 2002–2023. Basemap from Climate Data Toolbox[51], created using Matlab R2021a.

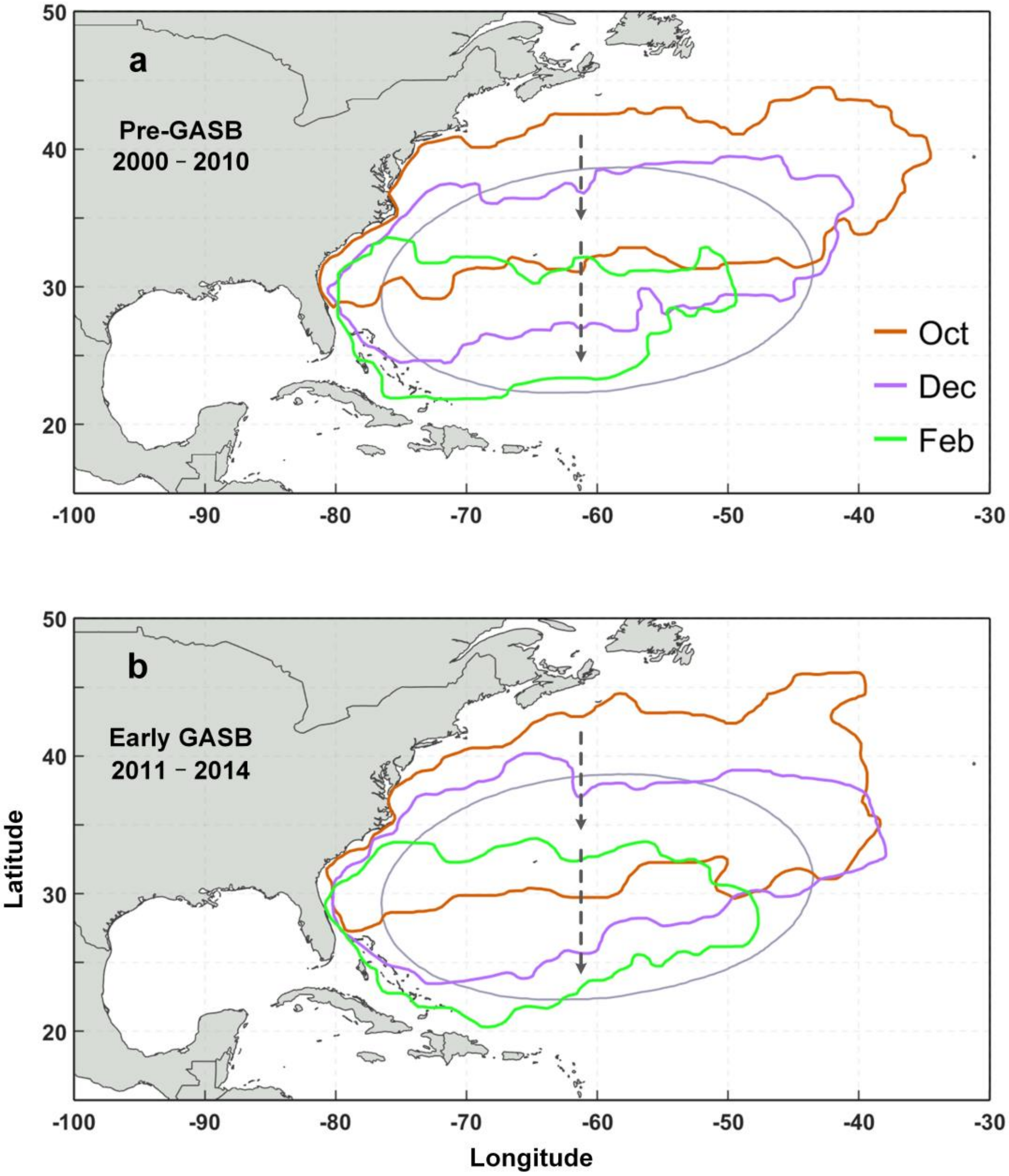

Supplementary Fig. 2 | Southward movement of *Sargassum* in the Sargasso Sea and its surrounding waters during fall and winter (October, December, and February). (**a**) and (**b**) are shown for the pre-GASB era (2000–2010) and early GASB period (2011–2014), respectively. These color-coded *Sargassum* boundaries were extracted from the monthly climatological distribution maps of *Sargassum* density shown in Extended Data Figs. 1,2. The grey arrows indicate the southward movement of *Sargassum*. Basemap from Climate Data Toolbox[51], created using Matlab R2021a.

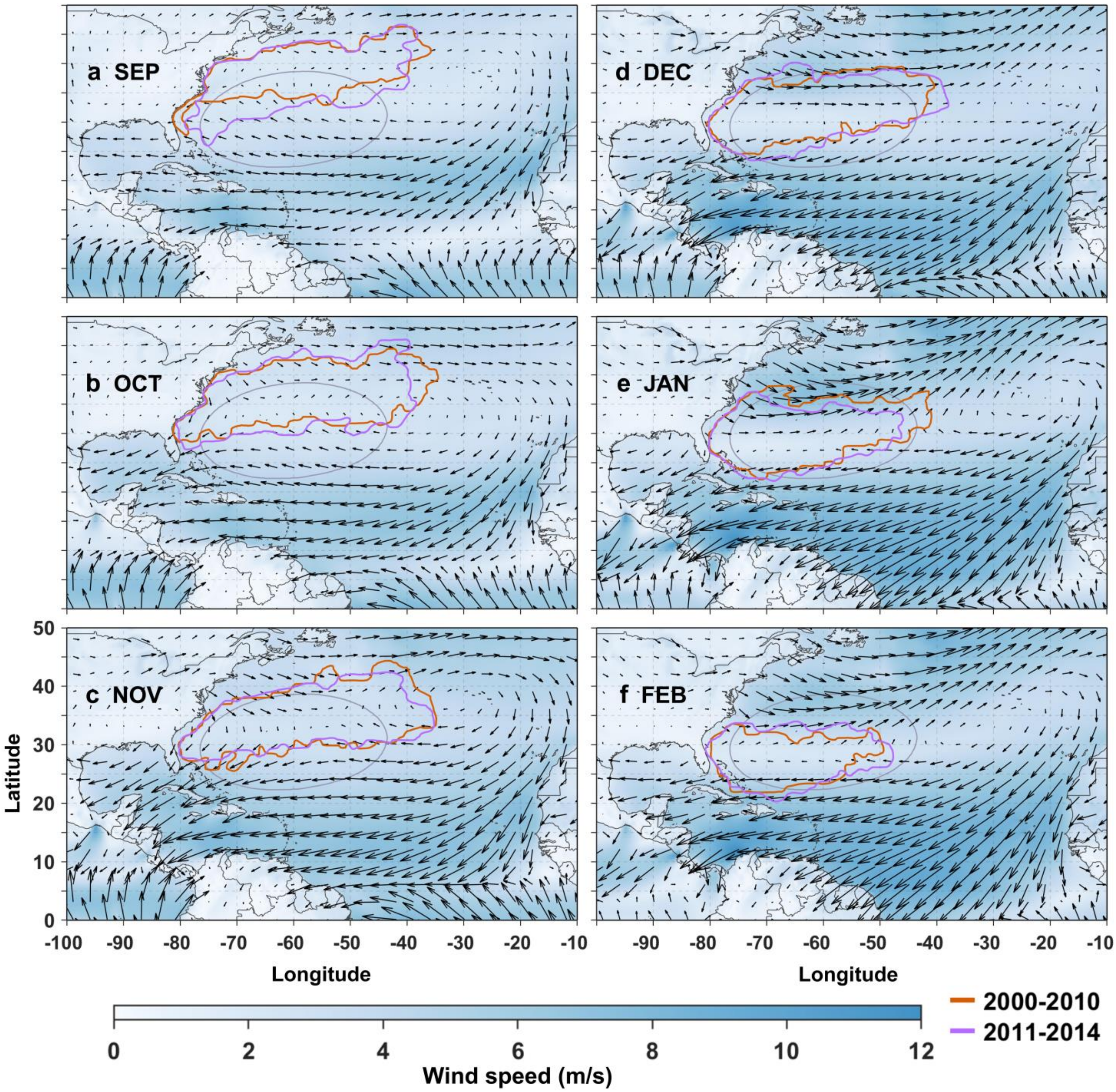

Supplementary Fig. 3 | Monthly climatological distributions of CCMP winds in the Gulf of Mexico, Caribbean Sea, and the North Atlantic between 2000 and 2023. The black vectors represent the direction of winds, and the arrow sizes and color scales indicate the wind speeds. The solid orange and purple curves in each subplot represent the *Sargassum* boundaries for the pre-GASB era (2000–2010) and early GASB period (2011–2014), respectively. Basemap from Climate Data Toolbox[51], created using Matlab R2021a.

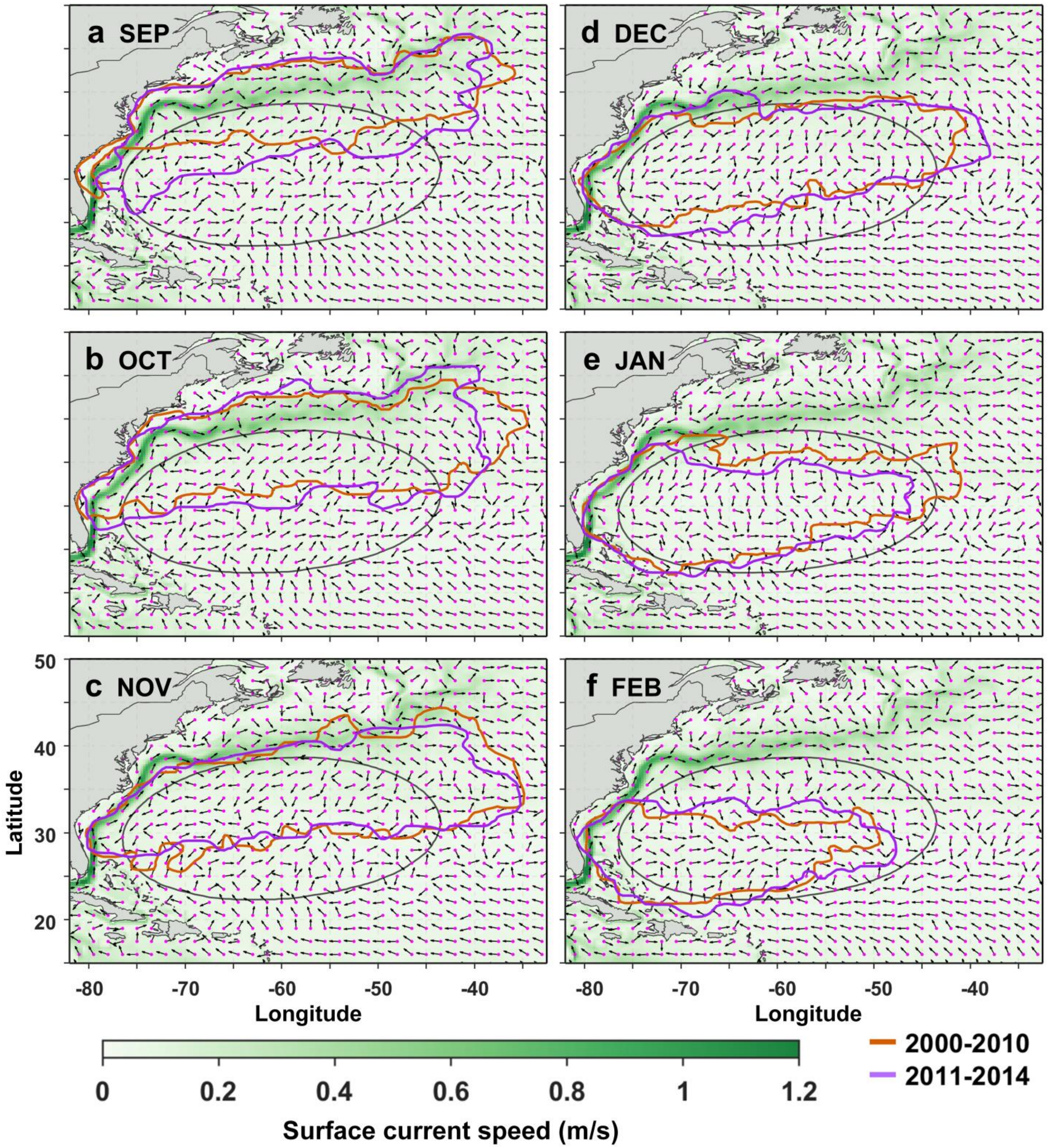

Supplementary Fig. 4 | Monthly climatological distributions of ORAS5 surface currents in the North Atlantic between 2000 and 2022. The black vectors represent the direction of surface currents, and the color scale indicates current speeds. The solid orange and purple curves in each subplot represent the *Sargassum* boundaries for the pre-GASB era (2000–2010) and early GASB period (2011–2014), respectively. Basemap from Climate Data Toolbox[51], created using Matlab R2021a.

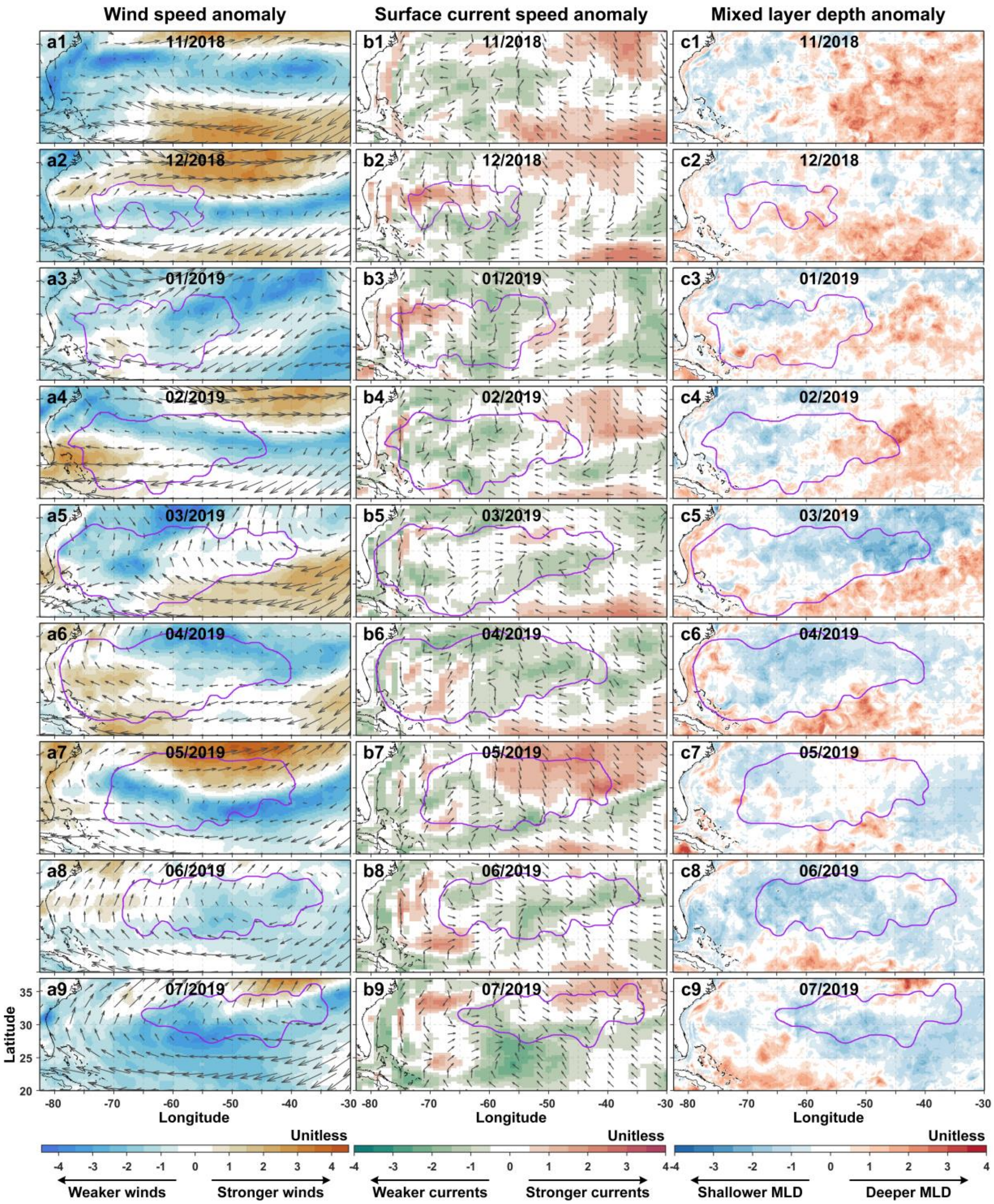

Supplementary Fig. 5 | Environmental conditions associated with the 2019 anomalous *Sargassum* bloom in the Sargasso Sea and its surrounding waters. Color scales in the left, middle, and right panels show monthly anomalies in CCMP wind speeds, NCEP-GODAS surface current velocities, and Global-HYCOM MLDs relative to the long-term monthly climatology, respectively. At each pixel, these anomalies were normalized by the corresponding standard deviation of monthly mean data in each climatological month. In the left and middle panels, the black vectors indicate wind and surface current directions, respectively. In each subplot, the purple curve indicates the boundary of the *Sargassum* patterns shown in Extended Data Fig. 7. Basemap from Climate Data Toolbox[51], created using Matlab R2021a.

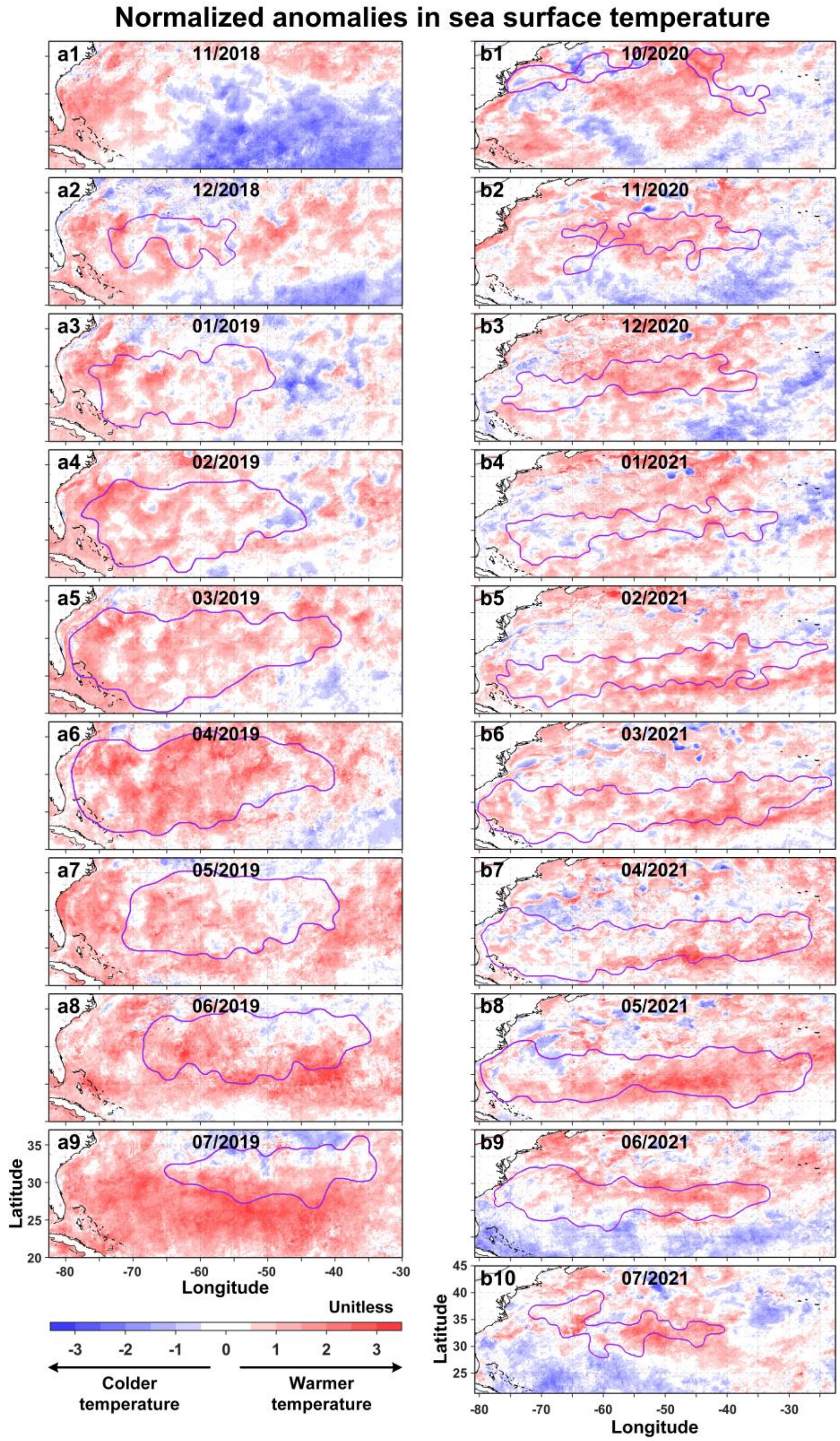

Supplementary Fig. 6 | Normalized monthly SST anomalies (unitless) for the Sargasso Sea and its surrounding waters during November 2018–July 2019 and October 2020–July 2021. The monthly SST anomalies are referenced to the long-term monthly climatology of SST between 2002 and 2023, and at each pixel, they were then normalized by the corresponding standard deviation of monthly mean SST in each climatological month. In each subplot, the purple curves indicate the boundary of *Sargassum* patterns in each corresponding month. Basemap from Climate Data Toolbox[51], created using Matlab R2021a.

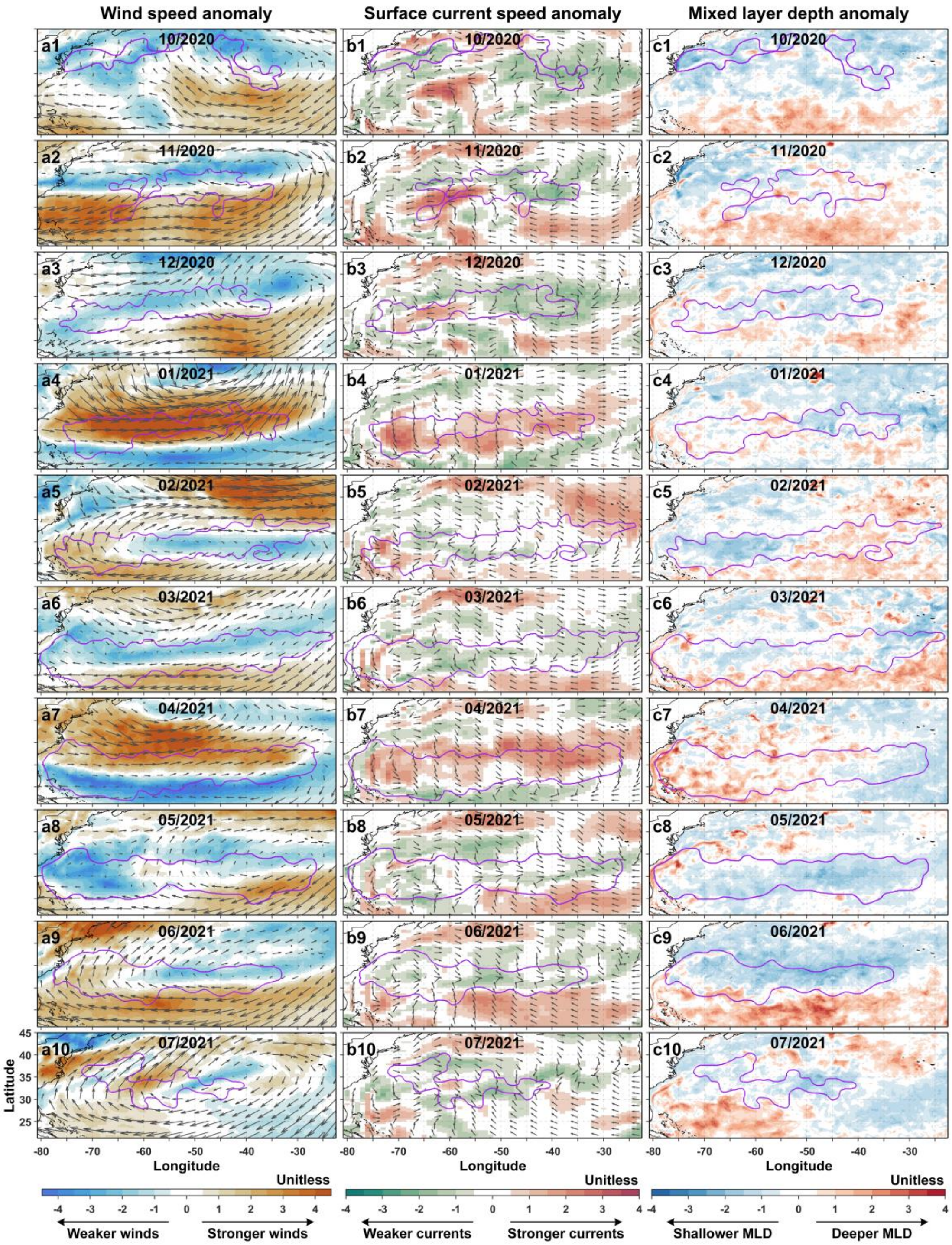

Supplementary Fig. 7 | Environmental conditions associated with the 2021 anomalous *Sargassum* bloom in the Sargasso Sea. Color codes in the left, middle, and right panels show monthly anomalies in CCMP wind speeds, NCEP-GODAS surface current velocities, and Global-HYCOM MLDs relative to the long-term monthly climatology, respectively. At each pixel, these anomalies were normalized by the corresponding standard deviation of monthly mean data in each climatological month. In the left and middle panels, the black vectors indicate the wind and surface current directions, respectively. In each subplot, the purple curve indicates the boundary of the *Sargassum* patterns shown in Extended Data Fig. 9. Basemap from Climate Data Toolbox[51], created using Matlab R2021a.

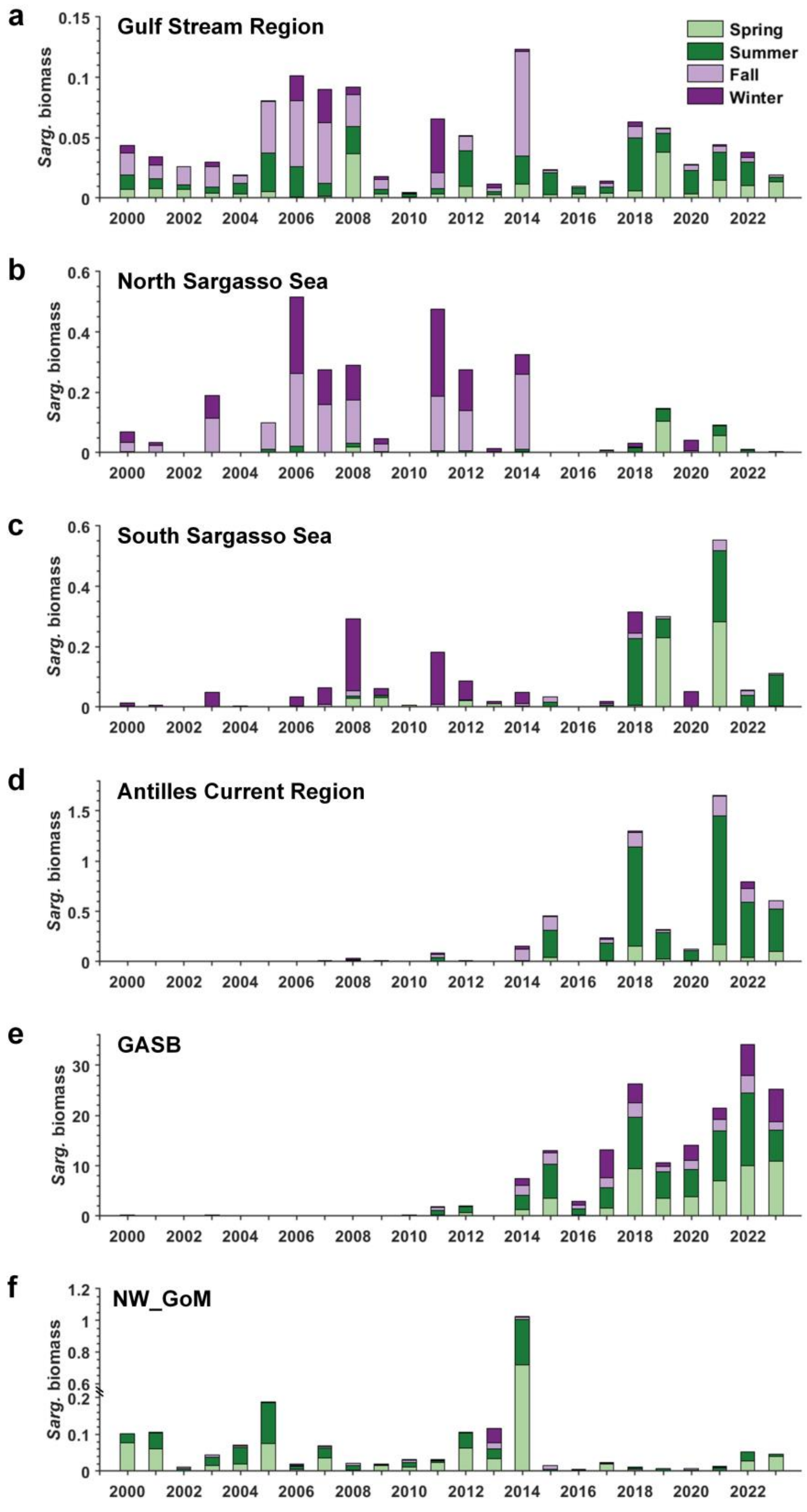

Supplementary Fig. 8 | Long-term seasonal changes in mean *Sargassum* biomass (in million tons) in six regions during 2000–2023 as observed by satellites. Note that the y-axes have different limits across the panels.

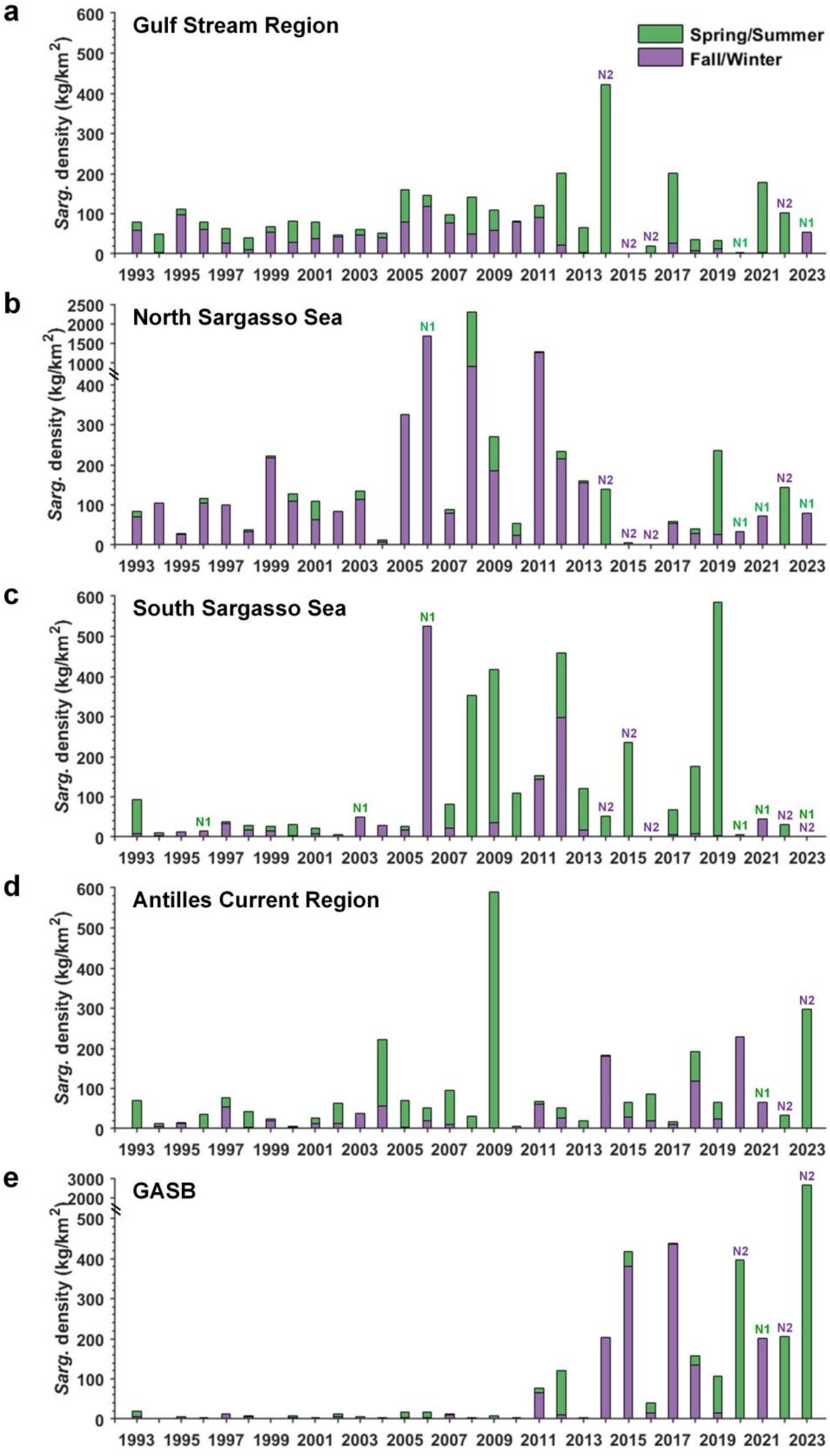

Supplementary Fig. 9 | Long-term seasonal changes in weighted mean *Sargassum* density in five regions during 1993–2023 as observed in the field. In each panel, "N1" indicates no *in situ* data in spring/summer, while "N2" means no *in situ* data in fall/winter.

Supplementary Table

Supplementary Table 1 | Multiple datasets for investigating potential environmental impacts on *Sargassum* spatiotemporal changes in the SS and adjacent waters. Note that these data products all have a monthly temporal resolution.

Environmental variable	Data source	Timespan	Spatial resolution (lat × lon)
SST	MODIS Aqua (https://oceancolor.gsfc.nasa.gov/)	2002–2023	4 km × 4 km
Chl-a concentration	MODIS Aqua (https://oceancolor.gsfc.nasa.gov/)	2002–2023	4 km × 4 km
Wind	REMSS CCMP V3.1 (https://www.remss.com/measurements/ccmp/)	2000–2023	0.25° × 0.25°
Surface currents	ECMWF ORAS5 (https://doi.org/10.48670/moi-00024)	2000–2022	0.25° × 0.25°
Surface currents	NCEP GODAS (https://psl.noaa.gov/data/gridded/data.godas.html)	2000–2023	1/3° × 1°
MLD	Global HYCOM (https://sites.science.oregonstate.edu/ocean.productivity/1080.by.2160.8day.inputData.php)	2000–2023	1/12° × 1/12°
MLD	Argo profiling floats (https://www.jamstec.go.jp/argo_research/dataset/milagpv/mila_en.html)	2001–2022	2° × 2°

Note. SST: sea surface temperature; Chl-a: Chlorophyll-a; MLD: mixed layer depth; MODIS: Moderate Resolution Imaging Spectroradiometer; REMSS: Remote Sensing Systems; CCMP: Cross-Calibrated Multi-Platform; NCEP: National Centers for Environmental Prediction; GODAS: Global Ocean Data Assimilation System; HYCOM: Hybrid Coordinate Ocean Model. For both MLD products, MLD is defined as the depth where the potential density is 0.03 kg/m^3 denser than at 10 m. This study utilized two surface current products: NCEP GODAS for large-scale current patterns (Supplementary Figs. 5,7) and ECMWF ORAS5 for smaller-scale current patterns (Supplementary Fig. 4). Similarly, two MLD products were employed: Argo-based MLD for climatological analysis (Fig. 3d and Supplementary Fig. 1) and HYCOM MLD for analyzing the anomalous events in 2019 and 2021 (Supplementary Figs. 5,7).

Supplementary Video 1 Caption

Animation of the monthly distribution of mean areal density of *Sargassum* in the Gulf of Mexico, Caribbean Sea, and the North Atlantic Ocean during 03/2000–02/2024.
